# Perceived Partner Responsiveness Forecasts Behavioral Intimacy as Measured by Affectionate Touch

**DOI:** 10.1177/0146167221993349

**Published:** 2021-03-19

**Authors:** Tatum A. Jolink, Yen-Ping Chang, Sara B. Algoe

**Affiliations:** 1 The University of North Carolina at Chapel Hill, USA

**Keywords:** affectionate touch, close relationships, perceived partner responsiveness, intimacy

## Abstract

Affectionate touch is an important behavior in close relationships throughout the lifespan. Research has investigated the relational and individual psychological and physical benefits of affectionate touch, but the situational factors that give rise to it have been overlooked. Theorizing from the interpersonal process model of intimacy, the current studies tested whether perceived partner responsiveness forecasts affectionate touch in romantic couples. Following a preliminary integrative data analysis (*N* = 842), three prospective studies use ecologically valid behavioral (Studies 1 and 2) and daily (Studies 2 and 3) data, showing a positive association between perceived partner responsiveness and affectionate touch. Furthermore, in Study 3, we tested a theoretical extension of the interpersonal process of intimacy, finding that affectionate touch forecasts the partner’s perception of the touch-giver’s responsiveness the next day. Findings suggest affectionate touch may be an untested mechanism at the heart of the interpersonal process of intimacy.

Affectionate touch is an important behavior for establishing and maintaining social bonds. It occurs frequently within close relationships, from infancy through older adulthood. It is defined as warm physical contact that communicates fondness and positive regard, as well as love and support (Jakubiak & Feeney, 2017). Across the lifespan, touch manifests in many ways, such as cuddling babies, kissing a romantic partner, and hugging a friend (Gulledge et al., 2004). Empirical work in relationship science has established affectionate touch as a common, normative behavior within close romantic relationships (Jones & Yarbrough, 1985; Suvilehto et al., 2015; van Anders et al., 2013) that can be distinct from sexual touch (Gulledge et al., 2004), and promotes individual and relational well-being (Jakubiak & Feeney, 2017).

However, what gives rise to affectionate touch within romantic relationship remains an open question. The current research investigates one theoretically plausible precursor to affectionate touch: perceived partner responsiveness. Demonstrating responsiveness during interpersonal interactions is one way to express care and support for another person (Reis & Shaver, 1988); the extent to which that responsiveness is perceived may then translate to enhanced psychological intimacy for the perceiver (Laurenceau et al., 1998). In turn, we expect this increased psychological intimacy to manifest as increased behavioral intimacy, as measured by affectionate touch, toward the partner who is perceived as responsive. After a preliminary concurrent analysis, we prospectively test the association between perceived partner responsiveness and ecologically valid affectionate touch in three studies of romantic couples.

## Affectionate Touch

While a host of behaviors can be considered touch (Jones & Yarbrough, 1985; Pisano et al., 1986), touch is only affectionate if it is implicitly meant to communicate love, care, and affection to the person receiving it (Floyd, 2006). Warm interpersonal contact like holding hands, hugging, kissing, and lying close together are prototypical examples of affectionate touch. Affectionate touch is common; touch is prevalent across culture (Andersen, 2011; DiBiase & Gunnoe, 2004), gender (Miller et al., 2014; Stier & Hall, 1984) and context (e.g., during mutual grooming, Nelson & Geher, 2007; between athletic teammates, Anderson & McCormack, 2015). However, affectionate touch most commonly occurs within close relationships, such as between family, close friends and romantic relationships (rather than between acquaintances or strangers; Jakubiak & Feeney, 2017; Jones & Yarbrough, 1985).

## Affectionate Touch in Close Relationships

Touch is a social behavior (Dunbar, 2010) and serves an important function for social bonding (Algoe & Jolink, 2020; Harlow, 1971). Beginning with the first prototypical “bond,” affectionate touch is a pathway through which caregivers (e.g., mothers) develop an intimate bond with their infants (e.g., secure attachment, Ainsworth et al., 1978; Bowlby, 1973). Much work has investigated the role of affectionate touch in child rearing and development (Ferber et al., 2008; Field, 2014; Hertenstein, 2002; Montagu, 1971), while the benefits of the receipt of affectionate touch within close adult relationships have been the focus of recent exploration in relationship science (for review, see Jakubiak & Feeney, 2017). In their review, Jakubiak and Feeney (2017) outline a framework of theoretical mechanisms through which receiving affectionate touch may be associated with relationship quality.

One possible reason affectionate touch receipt may be integral to romantic relationships, specifically, is its role in promoting intimacy. The experience of receiving touch is often perceived as intimate and the relationship therein is perceived similarly (Burgoon et al., 1984; Debrot et al., 2013; Floyd et al., 2005; Hertenstein et al., 2006; Johnson & Edwards, 1991; Mackey et al., 2000; Pisano et al., 1986; Thayer, 1986). Because social bonds are crucial to survival, affectionate touch may be a fundamental behavior necessary for strengthening such bonds—such as romantic relationships—by way of promoting intimacy.

Consistent with this theory, affectionate touch is also associated with long-term relationship outcomes. Affectionate touch receipt is positively associated with greater overall relationship satisfaction (Bell et al., 1987; Gulledge et al., 2003; Muise et al., 2014). Moreover, accumulating evidence suggests that in the moment, receiving touch promotes attachment security (Jakubiak & Feeney, 2016), helps to regulate emotions by enhancing positive affect (Debrot et al., 2013) and signals closeness or a desire for closeness (Ben-Ari & Lavee, 2007). Notably, most empirical work has focused on outcomes of those *receiving* affectionate touch.

Other research has explored individual and cultural characteristics that may make someone more likely to prefer receiving touch, such as attachment orientation (e.g., Brennan, Wu, & Loev, 1998; Chopik et al., 2014), gender (e.g., Johnson & Edwards, 1991; Stier & Hall, 1984), age (Heiman et al., 2011) and culture (e.g., dominance, DiBiase & Gunnoe, 2004; contact versus noncontact societies, Remland et al., 1995; Western versus East Asian countries, Suvilehto et al., 2019). While individual and cultural moderators help to explain *for whom* affectionate touch may occur and thus, provide the most relationship benefits, existing literature on affectionate touch does not reveal *why* that touch occurs, and specifically, the underlying situational precursors to the act of affectionate touch itself. That is, rather than focus on the recipient of touch, like prior research, we are interested in the touch-giver and what prompts their affectionate touch behavior. By focusing on a step earlier in the interpersonal process to consider possible situational or behavioral antecedents to affectionate touch provision, the field can begin to understand the conditions under which affectionate touch occurs. As such, beyond scholarly advances, this work could have translational impact.

## One Key Context: Moments of Intimacy

We propose one situation that may give rise to affectionate touch is when one romantic partner has already created a moment of intimacy within a dyadic interaction. Broad consensus in the close relationship literature is that one’s demonstration of responsiveness to a partner’s needs creates a feeling of intimacy for that partner, as outlined in the interpersonal process model of intimacy (Reis et al., 2004; Reis & Shaver, 1988). For example, when Issa tells her partner, Gene, about a stressor at work, Gene has an opportunity to respond to Issa’s disclosure. Issa’s perception of Gene’s response (e.g., was Gene supportive?) and the extent to which Issa interprets that interaction as intimate depend on whether Issa feels understood, validated, and cared for by Gene. Being responsive to the needs of a partner can be signaled in a variety of diverse situations, including during gratitude expressions (e.g., Algoe et al., 2013), positive event disclosures (e.g., capitalization, Gable et al., 2006), and negative event disclosures (Maisel et al., 2008). However, regardless of context, in situations when Issa perceives Gene as being truly responsive, it translates to feelings of psychological intimacy (Laurenceau et al., 1998; Reis et al., 2004).

The interpersonal process model of intimacy suggests that Issa’s perception of Gene’s responsiveness also translates to Issa’s behavior back toward Gene (Reis, 2007; Reis et al., 2004). Other researchers have documented behavioral outcomes after perceiving a partner’s responsiveness, such as proximity-seeking via approach behaviors toward a robot (Birnbaum, Mizrahi, et al., 2016) and greater expression of anxiety toward a partner during a stressful situation following manipulated thoughts about that partner’s responsive behavior (Ruan et al., 2020). In the current investigation, we focus on affectionate touch as another behavioral outcome of perceiving a partner as responsive. Our focus on affectionate touch is a novel contribution to the literature for at least two reasons. First, in their groundbreaking model, Reis and Shaver (1988) call out touch, specifically, as a behavioral mechanism within the intimacy process. Moreover, there is now strong consensus in the field, reviewed above, that affectionate touch is a behavior that is perceived as intimate (Burgoon et al., 1984; Debrot et al., 2013; Floyd, 2006; Hertenstein et al., 2006; Johnson & Edwards, 1991; Mackey et al., 2000; Pisano et al., 1986; Thayer, 1986). As such, although we do not measure intimacy in this article, we situate affectionate touch within the intimacy process model in our theorizing, considering it an instantiation of behavioral intimacy.^1^

Specifically, this work focuses on this theoretically derived but understudied first step of the mechanistic model in which perceiving a partner’s responsiveness to the self—that is, perceiving that person as more understanding, validating, and caring of the self—will translate to the perceiver’s greater likelihood of affectionately touching that partner, thereby theoretically converting perceived psychological intimacy (measured in perceived responsiveness) to enacted behavioral intimacy (measured by affectionate touch, Hypothesis 1). Second, consistent with the assumption that these are iterative interpersonal processes (Reis & Shaver, 1988), we predict the participant’s affectionate touch then feeds forward to predict the partner’s perception of the touch-giver’s responsiveness, which we test in Study 3 (Hypothesis 2).

## The Current Research

In the present investigation, we used multiple methods, with a strong emphasis on ecological validity, to triangulate evidence regarding our theorized path from perceiving a partner as responsive to affectionate touch. We did this using four dyadic data sets (*N* = 824). First, to better understand the general association between perceiving a partner as responsive and frequency of affectionate touch, we ran an Integrative Data Analysis as a Preliminary Study. We pooled data using the same two standardized measures across four existing data sets (*N* = 824) to provide an initial concurrent test of the hypothesized association and a robust estimate of the effect. Then, Studies 1, 2, and 3 involved prospective tests of the hypothesized association in three of those independent samples of romantic couples. In the first behavioral test of the theorical model in action, Study 1 used general perceived partner responsiveness to forecast moments of affectionate touch in daily life, using spontaneously reported behavior from 14 nightly reports. In a strong test of Hypothesis 1, in Study 2, we zoom in to the situation of interest, providing a clear initial test of whether responsiveness perceived in one laboratory task forecasts the perceiver’s touch of the partner in the next task, during a single lab visit. Study 3 used standardized scales from 28 nightly reports from both members of the dyad to test whether within-person variability in perceptions of partner responsiveness predicts greater affectionate touch of that partner that day, parsing within- and between-person variance. We also used this study to test a theoretical extension of the interpersonal process we are proposing. Due to past research implicating touch in the intimacy process (Debrot et al., 2012, 2013; Reis & Shaver, 1988), we specifically test whether one’s affectionate touch one day would subsequently forecast the *partner’s* perception of the toucher’s responsiveness the next day, that is, documenting the intimacy cycle (Hypothesis 2).

Because this study’s definition of affectionate touch does not require it to be sexual in nature (e.g., intercourse), and the fact that sex may be driven by a host of other factors (Birnbaum, 2010; Buss & Schmitt, 1993; Davis et al., 2004; Regan & Berscheid, 2001), in all possible studies (except Study 1, elucidated below) we take the conservative approach of operationalizing affectionate touch as *excluding* sex to more carefully focus on the types of affectionate touch that momentarily facilitate relationships in everyday life. In addition, in Studies 1 and 2, we wanted to address the possibility of a third variable, specifically, a dispositional or relational orientation, that could explain both greater perceived partner responsiveness and affectionate touch. Secure attachment, which is a trait-level orientation that relationship partners are and will be available and responsive (Mikulincer & Shaver, 2007; controlled in Study 1), communal strength, which is the unconditional motivation to meet a relationship partner’s needs (Mills & Clark, 1982; controlled in Study 2), and relationship satisfaction (controlled in Study 2) fit the bill. Then, moving away from a trait-level third variable to a situational variable that can vary within and between days and may also simultaneously explain both affectionate touch and perceptions of partner responsiveness, in Study 3, we controlled for daily relationship satisfaction.

## Preliminary Study

### Integrative Data Analysis: Testing the Basic Concurrent Association Across Four Samples

The first aim was to determine whether a positive association between perceived partner responsiveness and affectionate touch exists. Four dyadic data sets had been collected in the third author’s laboratory prior to the development of this hypothesis, yet contained general versions of each variable of interest: general perceptions of the partner’s responsiveness, and reports of the frequency of affectionate touch in the past month. This enabled us to take advantage of a powerful statistical technique, Integrative Data Analysis (Curran & Hussong, 2009; Hussong et al., 2013), to conduct a key initial test of the hypothesis. As implied by the label, this approach uses the raw data from all (four, in this case) data sets together; it is recommended over meta-analysis when possible because it uses rather than compresses the variance (e.g., into one effect size per study; Hofer & Piccinin, 2009) and does not rely on summarizing the data from only the mean and the variance. Using this method, we gain statistical power from the increased sample size of couples (824 members of 412 couples), and have representation across years, allowing an optimal statistical test of the key prediction (which is sensitive to detect a small effect |*ρ*| > .09 at power = 80% assuming independence among data).^2^

### Method

#### Participants and procedure

All participants were couples (*N* = 824 individuals) recruited from the community around Chapel Hill, North Carolina, by means of Craigslist volunteer ads and opt-in informational emails sent to university staff and students.^3^ Couples were eligible to participate if they had been in an exclusive romantic relationship for at least 6 months in Sample A and at least 1 year for Samples B, C, and D. In addition, they must have been living together for at least 6 months in Sample C. Across all four samples, couples had been together for on average 4.42 years, (*SD* = 4.75) with the majority of the total sample in an exclusive, committed relationship, that is, married (29.5%), engaged (8.6%), or dating exclusively (56.8%). Of the sample of couples, 63.1% were living together. The average participant was 26.39 years old (*SD* = 7.95, range = 55). Participants self-identified as White/Caucasian (64.6%), Black/African American (9.1%), East Asian (5.6%), South Asian (2.5%), American Indian or Alaskan Native (0.4%), or Other (7.2%). In a separate question, 6.4% of the sample identified as Hispanic. All measures were collected within baseline questionnaires of larger studies.

#### Measures

##### Perceived partner responsiveness

The extent to which participants feel their partner is generally responsive to them was assessed with an 18-item scale assessing understanding, validation, and caring (Reis, Crasta, et al., 2017). Items include, “My partner understands me,” “My partner is responsive to my needs,” and “My partner sees the ‘real’ me” measured on a 7-point Likert-type scale ranging from 1 (*not at all true/never*) to 7 (*very true/true all of the time*). Sample D used the 12-item scale (Reis et al., 2011). Although these use a different number of items across studies, given the fact that these scales effectively measure the same thing in our data and the relative homogeneity of these particular methods, we did not conduct a moderated nonlinear factor model (Curran et al., 2014) to extract a latent factor as one would in an IDA with more complicated methodologies across studies (Hussong et al., 2013). Instead, because we also did not have hypotheses about between-study mean-level differences, we standardized the variables by data set, which removed any between-data set effects while preserving the heterogeneity of the within-data set effect. The average reliability across the four samples was acceptable, α = .94; α_min_ = .92, and α_max_ = .95.

##### Affectionate touch

Participants reported on the frequency of affectionate touch with their partner in the past month (Light et al., 2005). The scale evaluates how frequently each behavior occurred in the past month from 0 (*never or almost never*) to 6 (*5 or more times a day*). The items were as follows: “how often do you hold hands with your spouse/partner?,” “how often do you sit close together or lie down close together with your spouse/partner while reading, watching TV or other leisurely activities?,” “how often do you give each other neck rubs, back massages or any other warm touching activities?,” “how often do you give your spouse/partner hugs lasting for more than a few seconds?,” and “how often do you kiss your spouse/partner?.” We computed the mean of the five items as our indicator of affectionate touch. For reasons stated above, all scales were standardized within the sample before merging and the average reliability across the four samples was satisfactory, α = .81; α_min_ = .78, and α_max_ = .84.

##### Sexual intercourse

An additional item, “how often do you and your partner have sex (intercourse)?,” which was measured on the same scale as the affectionate touch items, from 0 (*never or almost never*) to 6 (*5 or more times a day*), was included to test as a potential covariate.

### Results and Brief Discussion

Pooling the four dyadic data sets, we fit the harmonized data to a two-level (individual nested within couple) random-intercept model where affectionate touch was predicted by perceived partner responsiveness, data source was added as a four-category (Samples A–D) factorial predictor, and the interaction between data source and perceived partner responsiveness was tested (for the full analytic strategy for the IDA, see Online Supplemental material). The results support the hypothesis that perceived partner responsiveness is positively associated with affectionate touch. Specifically, the association was statistically significant across the merged data set, *b* = 0.29, *SE* = 0.07, *df* = 638.68, *t* = 4.00, *p* < .001, 95% CI = [0.15, 0.43], and not moderated by data source, *F*(3, 751.98) = 0.23, *p* = .88. This association explains 3% of the variance at the person level and 6% of the variance at the dyad level. We also tested a second model in which we controlled for sexual intercourse to address the possibility that in this romantic context, the affectionate touch might have been driven by having sex. The results confirmed that perceived partner responsiveness significantly positively predicted affectionate touch, *b* = 0.26, *SE* = 0.07, *df* = 647.43, *t* = 3.74, *p* < .001, 95% CI = [0.12, 0.40], above and beyond sexual intercourse, *b* = 0.25, *SE* = 0.03, *df* = 787.59, *t* = 7.42, *p* < .001, 95% CI = [0.19, 0.32], and the effect was again not moderated by data source, *F*(3, 756.51) = 0.42, *p* = .74.

Overall, this cross-sectional test of an association between generally perceiving one’s partner as responsive and the frequency of affectionate touch over the past month provides support for moving forward with the present investigation, which focuses on prospective tests of the hypothesis that perceiving responsiveness in the partner may prompt an individual’s affectionate touch toward that partner.

## Study 1: Prospective Link From General Perceptions of Responsiveness to Everyday Spontaneously Reported Affectionate Touch

In Study 1, we start with an ecologically valid assessment of the behavior of interest: affectionate touch spontaneously reported in mundane end-of-day descriptions of interactions with the partner across 14 days. Those daily responses, in which participants provided an open-ended description of a notable interaction with their partner each day, provide vibrant detail of not only how people recall their daily interactions with their partner, but importantly, how affectionate touch emerges spontaneously throughout everyday life. We took advantage of what participants described in those situations to code for the number of unique instances of affectionate touch described within the reports to test the present hypothesis. Specifically, we predicted general perceptions of partner responsiveness would predict more unprompted reports of affectionate touch behavior within the daily descriptions of notable partner interactions. Given the nature of the descriptions,^4^ in which it was not always obvious who initiated the touch, we opted to be inclusive of all mentions of affectionate touch during the interactions, regardless of partner, to take advantage of this rich initial opportunity to test links between perceived partner responsiveness and affectionate touch behavior in everyday life; the intimacy process model accounts for either explanation for effects (i.e., perceived responsiveness driving affectionate touch or partner’s affectionate touch driving responsiveness; Hypotheses 1 and 2). Because attachment styles are associated with touch (for review, see Jakubiak & Feeney, 2017), we also ran a model testing secure attachment as a possible dispositional third variable explanation.

### Method

#### Participants

Both members of 80 couples (*N* = 160 individuals) enrolled in a larger, 2-week study on “Everyday Experiences and Feelings of People in Romantic Relationships” (for more information, see Algoe & Fredrickson, 2019a). Sample size was determined for the goals of the original study. Couples had been together for approximately 4 years (*M* = 4.34, *range* = 6 months–35 years) and were either dating exclusively (48.8%) or committed for life (i.e., engaged or married; 41.9%). The majority of the couples identified as heterosexual (*N* = 77) while three identified as same-sex. Participants were between 18 and 57 years of age (*M* = 28.09, *SD* = 8.05). The majority self-identified as White/Caucasian (74.4%) with the remaining participants identifying as East Asian (1.9%), African American (12.5%), South Asian (2.5%) or Other Races/Ethnicities (5.0%). In a separate question, 3.8% participants identified as Hispanic.

A sensitivity analysis reveals the sample size (multiplied by number of nightly reports) is sensitive to detect a small effect|*ρ*| > .07 at power = 80% assuming no interdependence among data.

#### Procedure

Both members of the couple attended the initial lab session, where they completed a set of initial online questionnaires, participated in a series of lab tasks together, then received instructions about the 2-week nightly diary portion of the study they would complete before attending a second lab session. The focus of the present investigation is the self-reported general perception of responsiveness from the initial lab session (described and included in the IDA, Sample A; α = .92) and a novel nightly measure in which participants briefly described the most notable interaction with their partner that day.

Specifically, participants were asked to complete a 10-min questionnaire at the end of each day; they were asked to do so at about the same time every day. On average, individuals completed 11.63 nightly questionnaires in the 14-day study period (thus 1,860 reports of a possible 2,240), showing a high compliance rate (83.07%), and only five participants did not complete any nightly reports. The specific measure of interest from the nightly questionnaire was an open-ended response that participants gave to a prompt to briefly describe an interaction with their partner that “made the biggest impression today.” For this item, 380 reports out of the received 1,860 (79.57% compliance) did not contain any content and thus were not coded for analysis.

##### Spontaneously reported affectionate touch behavior

As described above, the prompt participants received did not explicitly instruct them to write about affectionate touch. The actual events participants wrote about may have been minor or major, positive or negative, but critically, upon reading the whole corpus, we noticed that the interactions with the partner that made the biggest impression contained several instances of explicit mention of affectionate touch. Hence, we coded the text for the number of spontaneous reports of affectionate touch. Two independent judges were trained to code the nightly data. After training, coders practiced by independently working through the same 150 responses to establish reliability (8% of the total nightly responses in the study). Then, the remaining 1,710 responses were independently coded by each rater. Throughout, all coders were unaware of the hypothesis.

To code for touch in the daily reports, coders were instructed: “Count each unique instance of affectionate touch mentioned in the nightly event description.” For instance, this participant’s response, “[my partner] came by work and saw me real quick. its always nice to see her in the middle of the day and give her a hug and kiss” would have been coded as a 2 (i.e., hug, kiss). Notably, some participants mentioned desiring affectionate touch from their partner and not receiving it. Any mention of a touch “deficit” was not included in the count. In addition, some phrases seemed highly likely to include affectionate touch, so were included, despite the fact that they also may have included sexual activity (e.g., “get intimate,” “spent the night together”); because of this, the count code also included explicit mentions of “sexual intercourse.” Verbatim instructions are in the Online Supplemental material.

Coders noted anywhere from 0 to 5 instances of affectionate touch (ICC = .89) in a given entry. For 4.2% of responses (*N* = 79), coders had different counts of affectionate touch and a third rater (the first author) arbitrated based on fidelity to the definition.

*Sexual Intercourse*. The presence (coded 1 vs. absence = 0) of sexual intercourse was also coded separately to be used as a control variable. See the Online Supplemental material for details of that coding procedure.

*Attachment*. The 35-item Experiences in Close Relationships Scale (Brennan, Clark, & Shaver, 1998) measured anxious (e.g., “I worry about being alone.”) and avoidant (e.g., “I try to avoid getting too close to my partner”) attachment dimensions on a scale from 1 = *strongly disagree* to 7 = *strongly agree*. Reliability was acceptable for both anxious attachment and avoidant attachment, α_anx_ = .90, α_avoid_ = .91, and the two dimensions were positively correlated in the sample, *r* = .28, *p* < .001.

### Results

#### Planned statistical models

We used two-level cross-classified models in which partners are nested within dyads, then crossed by report days because both partners filled out reports on the same days (Kenny et al., 2006). We allowed intercepts to randomly vary, while slopes of the day-level predictors were modeled as fixed effects. We had no a priori hypothesis about gender, so dyads were treated as indistinguishable and we used a diagonal residual matrix.^5^

#### Validation: Daily affectionate touch

As one assessment of the validity of the behavioral codes, we conducted a multilevel linear model using baseline general affectionate touch in the prior month (see IDA) to predict the daily affectionate touch count code; the positive association was significant, *b* = 0.03, *SE*, = 0.01, *t* = 2.43, *p* = .02, 95% CI = [0.01, 0.05].

#### Affectionate touch in everyday life

We next conducted a two-level cross-classified linear model wherein each person’s daily-level affectionate touch was predicted by his or her individual-level, general baseline perceptions of partner responsiveness. Supporting the theoretical model, the results showed that those who reported perceiving higher responsiveness in their partners in general at study entry reported more affectionate touch in their subsequent everyday interactions with the partner, *b* = 0.03, *SE* = 0.02, *df* = 395.51, *t* = 2.04, *p* = .043, 95% CI = [0.00, 0.07]. We then controlled for explicit mention of sexual intercourse and results remain consistent, with general perceptions of partner responsiveness remaining positively associated with more affectionate touch in everyday life, *b* = 0.03, *SE* = 0.01, *df* = 292.82, *t* = 2.12, *p* = .035, 95% CI = [0.00, 0.06], and controlling for sexual intercourse, *b* = 1.24, *SE* = 0.06, *df* = 1856.00, *t* = 22.05, *p* < .001, 95% CI = [1.13, 1.35]. General responsiveness predicts 0.05% of the variance at the person level and 4.53% of the variance at the couple level.

#### Analyses of alternative hypotheses

Addressing the possibility that these findings could be explained by more securely attached individuals having both greater general perceptions of partner responsiveness and engaging in more affectionate touch in everyday life, our additional analysis controlling for attachment styles documented that general perceptions of partner responsiveness remained a significant predictor of spontaneously reported touch, whereas neither attachment anxiety nor avoidance predicted touch in this model. Full results are presented in the Online Supplemental material.

### Brief Discussion

Study 1 provides the first test of which we are aware linking perceived partner responsiveness with unprompted, spontaneous reports of affectionate touch behavior in everyday life, thereby substantially adding to the slim literature on this topic (e.g., Debrot et al., 2013). One limitation of the coding, however, is that it does not cleanly disentangle the direction of the association (i.e., Hypothesis 1 or Hypothesis 2) due to ambiguity in who initiated the touch in some reports as well as the fact that about one third of responses included mention of partner-initiated affectionate touch. Therefore, in Study 2, we turn to direct observations of the touch-giver.

## Study 2: Prospective Link From In Situ Perceptions of Responsiveness to Subsequent Affectionate Touch in Lab

Study 2 builds on the findings from Study 1 by zeroing in on touch *provision*, cleanly testing whether situational perceptions of a partner’s responsiveness predicts in-the-moment observations of touch (Hypothesis 1). This study provides a prospective test of our hypothesis, testing the link from one situation to the next. We also focus on an objective measure of touch, using observed behavior in a videorecorded laboratory conversation. Specifically, we operationalized perceived responsiveness in an initial situation by having participants rate their partner after the partner expressed gratitude to them in a standardized laboratory task (see Algoe et al., 2013). Then, we created an opportunity in the lab for partners to spontaneously touch. Specifically, after the partner expressed gratitude to the participant and the participant privately reported the partner’s responsiveness, we briefly separated them, then allowed the participant to rejoin the partner to “hang out” while experimenters ostensibly prepared the next portion of the study in a different room. Experimenters recorded the first 5 minutes of this reunion as participants were seated together on a couch. During this time, there was variability in affectionate touch, with some couples not touching at all to others who kissed or enacted idiosyncratic affectionate touches like foot rubs or sitting on laps.

Modeling our behavioral coding scheme on the coding used in Study 1, we coded these interactions for the participant’s affectionate touch of the responsive partner. Specifically, we coded the number of unique instances of affectionate touch demonstrated from the perceiver toward their partner. During the interactions, a subset of couples kissed. We were somewhat surprised, but also saw an opportunity: kissing is a well-documented display of affection in the United States (e.g., Hughes et al., 2007; Welsh et al., 2005), signifies social connection worldwide (Eibl-Eibesfeldt, 1970), and yet its presence in this laboratory context is not normative. As such, we view the presence of kissing as a strong signal of the construct of interest—behavioral intimacy as measured by affectionate touch. Thus, separately, we coded whether or not kissing occurred in the video to test whether the perceptions of partner’s responsiveness following the gratitude expression predicted a greater likelihood of kissing during the subsequent interaction.

Because communal strength can feed into perceptions of responsiveness (Lemay & Clark, 2008; Reis et al., 2004) and relationship satisfaction could explain between-person differences in situational perceptions of responsiveness and affectionate touch, we control for communal strength and separately, relationship satisfaction, as dispositional and relational third variable explanations.

### Method

#### Participants

Both members of 129 heterosexual couples (*N* = 258 individuals) who were romantically involved for at least 1 year and living in the area surrounding Chapel Hill, North Carolina, were recruited to participate in the study. Most couples were exclusively dating (76.7%) and 23.3% reported being engaged, married or “living as married.” Fifty-six of the 129 couples (43.4%) were living together at the time of the study. Participants ages ranged from 18 to 50 (*M* = 23.7, *SD* = 5.64) and most identified as Caucasian (70.9%). The rest identified as East Asian (11.2%), African American (7.4%), South Asian (4.7%), or Other racial backgrounds (5.8%). In a separate question, 9.9% of the sample identified as Hispanic. Sample size was determined a priori to test a different hypothesis (see Algoe et al., 2016). However, it was estimated that the 125 participant videos (four participants did not produce an analyzable video, explained below) still allowed us to detect an effect as small as|*ρ*| > .24 at 80% power assuming no interdependence among data, which is a purposefully conservative assumption.

#### Procedure overview

Both members of the couple attended the lab together as part of a larger study (Algoe, 2019). This aspect of the procedure was designed to address the impact of hearing an expression of gratitude (i.e., perceiving responsiveness of the Expresser, the primary measure in a larger program of research; for example, Algoe et al., 2013) on behavior within a subsequent interaction. To that end, one member of the couple was randomly assigned to express gratitude to the other; for clarity, we will refer to these dyad members as the *Expresser* and the *Target* of the expression, respectively. The Target’s perception of and behavior toward the Expresser are the focus of this investigation.

After the gratitude expression task, the Target privately rated their perceptions of the Expresser’s responsiveness within a brief questionnaire. Experimenters then used a cover story to have the Target leave the primary lab room and relocate to a room across the lab suite, rearrange the primary lab room so the Expresser was sitting on a couch with no other chairs in the room, and disappear into the control room within the lab suite; then, when the Target returned to the primary lab room of his or her own volition when done with the minor task, the experimenters videorecorded the first 5 min of the couple’s reunion on the couch. The video camera was on the wall directly across from the small couch, so the couch and participants were fully framed within the image. The experimenters re-entered the room after recording for 5 min to administer the final tasks of the session (unrelated to the current investigation) and to debrief the couple before they left the lab.

##### Perceived Expresser responsiveness after expressed gratitude conversation

Instructions for selecting the grateful event and having the expressed gratitude conversation followed the standard paradigm (Algoe et al., 2013), with the caveat that just prior to the conversation, in a different room across the lab suite, the Expresser had been privately given additional guidance about *how* to express, without the Target’s knowledge. The Expresser was randomly assigned to one of the two instruction conditions; that experimental manipulation, the fact that it had no effect on the outcome of interest—Target’s perceived partner responsiveness—and why that may have been, are all extensively documented in a prior publication (Algoe et al., 2016). Here, our interest was in whether perceptions of partner responsiveness (which were not affected by the manipulation) forecasted a subsequent behavior, so we collapse across condition in analyses. Nevertheless, because the instructions may have influenced the Expresser’s behavior in the interaction, we control for condition in analyses (coded as either 0 = positive active control or 1 = other-praising).

Immediately following the conversation, Targets rated their perceptions of the Expresser’s responsiveness during the previous interaction using a 10-item situational measure of responsiveness (Gable et al., 2006). The scale ranged from 0 (*not true at all/never true*) to 6 (*very true/true all of the time*), with 3 representing (*moderately true/true all of the time*), and higher numbers representing greater perceived partner responsiveness during the gratitude expression task. The reliability the Target’s rating of these items was satisfactory (α = .94).

Responsiveness ratings were missing from one participant due to a procedural error. We also excluded three participants who had exceptionally low scores on this scale, consistent with and justified in a prior publication using these data (Algoe et al., 2016). For transparency, we present results including those participants in the Online Supplemental material.

##### Private leisure time

After completing the post-interaction measures, the couple was told that there would be about 20 min before the next lab task while the experimenters prepared some materials. The experimenter then used the stated gap of time as an “incidental” reason to ask for the Target’s help pilot testing some materials for a different study in another room, across the lab suite (see the Online Supplemental material for information on the task). The Target was informed that he or she could go back across to the primary lab room (where the partner was), whenever he or she was done. In reality, the pilot testing task was a ruse to get the Target out of the primary lab room.

After the Target left, the second experimenter went into the primary room, where the Expresser was, to rearrange it, stating that the two chairs and laptop tables were not needed anymore, and inviting the Expresser to move to the couch along the wall of the same room. After quickly moving everything to one side, incidentally leaving the couch as the only natural place for the Target to sit upon re-entering the room, the experimenter offered the Expresser a magazine while he or she waited, then left the room to join the first experimenter in a control room within the lab suite; once in the control room, experimenters closed their door to the rest of the suite to enhance participants’ perceived privacy.

The couch was directly across from a video camera that the participants were aware had been used to videorecord their prior gratitude conversation but was not mentioned again as the experiment continued. After the Target completed the ratings of pilot materials, he or she crossed the lab suite to re-enter the room with the Expresser, joining the partner on the couch to relax until the next part of the study. Experimenters recorded video for 5 min from the time the Target sat down on the couch with the Expresser. Videos were not obtained for four couples due to procedural errors.

*Behavioral Coding: Affectionate Touch*. Two trained coders separately watched the 5-min video without sound and coded it for the Target’s affectionate touch of the Expresser. The coders received thorough training that included 2 weeks of practice coding (15 videos) with regularly scheduled meetings to discuss and recalibrate codes. After reliable scores were reached, the two coders independently coded the 125 videos for the focal behavior: affectionate touch. Throughout the behavioral coding, coders were kept unaware of the hypothesis.

Affectionate touch was defined in the coding instructions as “warm physical contact that communicates fondness and positive regard, as well as love and support” that “can take many forms but some instantiations are hugging, kissing, stroking, and cuddling” and be done with “the hand, the whole body, or any other part such as head, foot, and lips.” Affectionate touch did not include functional touches, such as accidentally brushing hands when turning the pages of the magazine, or aggressive touches, such as intentionally hurtful, non-playful pinches, or pokes. The judges coded affectionate touch in two ways.

First, two coders documented each unique instance of when the Target touched the Expresser affectionately (ICC = .88), which created a count variable of the number of enacted affectionate touches from Target to Expresser during the 5 min. For example, if the Target (a) leaned into the crook of the Expresser’s open arm, during which he or she (b) patted the Expresser’s leg, (c) nudged the Expresser with his or her foot, and (d) patted the Expresser’s leg again, the coders would code the Target as having demonstrated affectionate touch four unique times during that set of behavior. Coders observed anywhere from 0 to 32 instances of affectionate touch within the 5-min period. A difference score was computed between the two coders. For any videos with a difference score of 3 or less, the two coder’s scores were averaged together. For 8% of videos (*N* = 10), coders reported a difference score of more than 3 and a third rater (the first author) arbitrated based on fidelity to the training instructions.

Second, coders also provided an overall affectionate touch *rating* for each video. Because results are similar and the touch count is the more objective of the two measures, we describe this rating measure and results in the Online Supplemental material to save space.

*Behavioral Coding: Kissing*. Separately, videos had been coded by a prior team for whether the couples kissed, and this included kisses that had been initiated by the Expresser (ICC = .97). Most of the kisses were brief, and although many were kisses on the partner’s lips, some were kisses on top of the partner’s head, on the partner’s shoulder, or on a hand. For the present investigation, the first author (unaware of participants’ ratings of responsiveness) eliminated instances where the only kiss within the video was obviously initiated by the Expresser, resulting in a code documenting any evidence of Target-initiated kissing within a video as indicating the presence (coded as 1) of this strong signal of behavioral intimacy; videos that did not include a kiss from the Target to the Expresser were coded as (0). These criteria eliminated six videos where the only kiss was initiated by the Expresser (coded as 0), leaving a total of 27 videos that included a kiss initiated by the Target.^6^

*Behavioral Coding: Expresser Engagement*. We observed variability in Expresser behavior upon Target’s re-entry to the room with the couch, including disengagement (e.g., Expresser had head buried in the magazine and didn’t look up). To address the possibility that the proposed effect on Target’s affectionate touch could be influenced by the Expresser’s recent behavior, rather than the Target’s perception of the Expresser’s responsive behavior in the prior situation, four coders rated how interested and engaged the Expresser was toward the Target from the time the Target entered the primary lab room until they sat down on the couch.

Expresser engagement was rated at the following levels: 1 = *disinterested* (i.e., unaware or disinterested in partner’s return); 2 = *acknowledgment* (i.e., looks up upon partner’s return); 3 = *interested and welcoming* (i.e., smiles or has look of anticipation upon partner’s return); 4 = *excited and very welcoming* (i.e., high levels of engagement upon partner’s return, such as continued positive facial expressions, making physical room on the couch, displaying welcoming arm gestures), *M* = 2.50, *SD* = 0.89. ICC for the four coders was .94. We control for Expresser engagement in all forthcoming analyses; results without this pre-planned covariate are in the Online Supplemental material.

*Communal Strength*. Using the Communal Strength Scale (Mills et al., 2004), participants responded to 10 items (e.g., “how easily could you accept not helping [*your partner*]?”) on a 10-point scale (1 = *not at all* to 10 = *extremely*). Reliability was acceptable, α = .84.

*Relationship Satisfaction*. Relationship Satisfaction was measured globally (e.g., “how much do you love your partner?”) with seven items on a seven-item Likert-type scale (Hendrick, 1988), α = .86.

### Results

#### Validation: Target affectionate touch

The correlations presented in Table 1, Row 5, indicate that the coded affectionate touch is consistent with the definition of affectionate touch: Target’s self-reported frequency of affectionate touch in the prior month (described and included in the IDA, Sample A α = .81) is positively correlated with each code of their behavior in the private leisure time interaction.

**Table 1.** Means, Standard Deviations, and Correlations Among All Study 2 Variables.

Measures	1	2	3	4	5	6	7
1. Self-reported perceived Expresser responsiveness following conversation	—						
2. Coded Target affectionate touch behavior	.22*	—					
3. Coded Target kissing	.28**	.69**	—				
4. Coded Expresser engagement	.09	−.004	.13	—			
5. Baseline self-reported frequency of affectionate touch in prior month	.23*	.27**	.28**	.04	—		
6. Baseline communal strength	.49**	.14	.03	.05	.28**	—	
7. Baseline relationship satisfaction	.42**	.06	.16	−.01	.35**	.35**	—
Mean	5.45	6.19	.22	2.50	4.39	7.38	6.29
*SD*	.58	7.01	.42	.69	.92	.90	.51
Min-max	[3.6, 6.0]	[0, 32]	[0, 1]	[1, 4]	[1.2, 6.0]	[4.4, 9.2]	[4.43, 7.0]

*Note.* SD = standard deviations.

* *p* < .05. **p < .01.

#### Affectionate touch behavior

Due to the distribution of the touch count, we conducted a linear regression using bootstrapped estimates (1,000 repetitions) of the confidence intervals to test whether perceptions of a partner’s responsiveness following a gratitude expression would prospectively predict affectionate touch in a subsequent interaction. As predicted, perceived Expresser responsiveness was positively associated with the target’s affectionate touch of the Expresser in the lab, *b* = 2.55, *B* = .22, *SE=* 0.98, *t* = 2.30, *p* = .016, bootstrapped 95% CI = [0.67, 4.50]. The bootstrapped estimate of the confidence interval did not include zero, thus supporting our prediction. Target’s affectionate touch was not significantly predicted by condition, *b* = 0.39, *B* = .03, *SE =* 1.29, *t* = 0.30, *p* = .76, bootstrapped 95% CI = [−2.06, 3.04], or Expresser engagement, *b* = −0.20, *B* = −0.02, *SE =* 1.03, *t* = −.21, *p* = .86, bootstrapped 95% CI = [−2.33, 1.81]. As Targets perceived their partner as more responsive in one situation, they engaged in a greater degree of affectionate touch toward their partner during “private time” in a later interaction.

#### Kissing

A multiple logistic regression model showed that standardized perceived Expresser responsiveness predicted a greater likelihood of kissing during private leisure time, *b* = 1.13, *SE* = 0.40, *df* = 1, *p* = .005, OR = 3.08, 95% CI = [1.42, 6.71], whereas kissing was not significantly predicted by condition, *b* = 0.42, *SE* = 0.47, *df* = 1, *p* = .37, OR = 1.53, 95% CI = [0.60, 3.86], or Expresser engagement, *b* = 0.54, *SE* = 0.35, *df* = 1, *p* = .12, OR = 1.72, 95% CI = [0.87, 3.42], due to the confidence intervals of these odds ratios including 1, indicating non-significance. After standardizing the responsiveness scale for interpretation, this translates to the following: a one-standard deviation increase in the Target’s perception of the partner responsiveness was associated with a 308% greater likelihood of kissing the Expresser in the subsequent interaction.

#### Analyses of alternative hypotheses

To test the possibility that a dispositional or relational third variable was driving the hypothesized effect, we also ran models controlling for general communal strength and global relationship satisfaction. The effect of perceived responsiveness following the gratitude expression remained significant in predicting both coded affectionate touch and kissing (tested separately), even when accounting for communal strength or relationship satisfaction (tested separately, neither of which significantly predicted affectionate touch). For space, we present full model results in the Online Supplemental material.

### Brief Discussion

In a tight prospective test of our hypothesis, the current evidence documents that perceiving responsiveness in the partner in one situation forecasts the participant’s greater affectionate touch of the partner in the next. We assessed affectionate touch behavior two ways: by counting all instances, and by observed kissing. Participants who perceived their partners as more responsive when expressing gratitude in the lab subsequently engaged in more overall spontaneous affectionate touch of their partner and specifically were more likely to kiss their partner when given an opportunity to spend private time with them later.

Other research demonstrates the power of perceived partner responsiveness—particularly after receiving an expression of gratitude—in shaping future relationship outcomes (Algoe et al., 2013, 2016). Here, we used one partner’s gratitude expression to conduct a time-sensitive test of whether in-the-moment perceptions of partner responsiveness would forecast greater likelihood of affectionate touch in a subsequent interaction.

One strength of this study is the use of coded behavioral demonstrations of affectionate touch. Investigations using real touch behavior—versus self-reports—are rare, and Study 2 demonstrates the prevalence and power of real-life affectionate touching. For example, one couple laid down to “spoon” each other on the couch, many participants stroked their partner’s head, one man gave his partner a foot rub. These moments of physical connection, which followed feeling more understood and cared for by their partners a few minutes prior, support our underlying theoretical assumption of a conversion of perceived psychological intimacy into behavioral intimacy. Furthermore, in one of the first tests examining the link between communal strength and affectionate touch behavior, we find these effects cannot be explained by communal strength or relationship satisfaction.

The affectionate touch coding focused only on the Target’s touch allowed for a precise test of our hypothesis, using a nice range of affectionate touch behaviors as well as one high-fidelity signal of affection in the lab setting, a spontaneous kiss.

## Study 3: Day-To-Day Variability in Perceptions of Responsiveness and Affectionate Touch

Thus far, evidence from concurrent and prospective analyses, as well as in-lab behavior and daily behavior, supports the hypothesis that perceived responsiveness forecasts affectionate touch. In Study 3, we use nightly reports from each member of the couple to illuminate the daily dynamics of this association. Across 28 days, participants reported their perception of their partner’s responsiveness and the affectionate touch they provided to their partner, both in the past 24 hr. With data that include both the independent and dependent variable each day, we are able to disaggregate the variance to (a) variance attributed across people—the between-person effects—and (b) variance-attributed within-person (Bolger & Laurenceau, 2013; Curran & Bauer, 2011). The between-person effect explains what happens, on average, across individuals, as has been our focus in the manuscript thus far. The within-person effect provides a tighter test of our hypothesis that situational variability in perceived partner responsiveness is significantly positively associated with situational *changes* in affectionate touch of that partner. Specifically, in the within-subjects model, we predicted that on days that Issa perceives Gene to be *more responsive than she usually does* (i.e., taking into account her own average), Issa would demonstrate greater affectionate touch toward Gene that same day, controlling for Issa’s affectionate touch the day before. By controlling for affectionate touch the previous day, we interpret effects as representing the change in affectionate touch that day.

**Figure 1.**
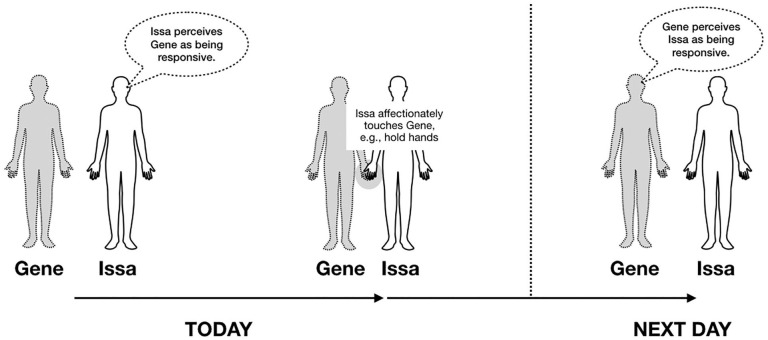
Conceptual model of two-step, recursive daily dyadic process.

Next, we test whether the affectionate touch Issa reports providing one day may then feed forward into Gene’s perception of Issa’s responsiveness on the next day (Hypothesis 2). We test this in a second model, disaggregating between- and within-person effects and controlling for perceived responsiveness of the touch-giver that present day (i.e., same day as Issa’s touch). See Figure 1 for the conceptual model of this two-step process.

We also attempted to rule out a fluctuating situational variable—daily relationship satisfaction—that could alternatively explain the hypothesized effects. For Hypothesis 1, we focused on participant’s relationship satisfaction. For Hypothesis 2, we ran two theoretically reasonable tests, one relevant to the independent variable and the other relevant to the dependent variable: (a) touch provider’s satisfaction on the day they touched and (b) touch receiver’s satisfaction on the day they perceived touch-giver’s responsiveness.

### Method

#### Participants

Both members of 53 heterosexual couples (*N* = 106 individuals) who had been cohabitating for at least 6 months and living in the area surrounding Chapel Hill, North Carolina, were recruited to participate in the study, “Understanding Romantic Relationships”; sample size was determined for the goals of the original study (Algoe & Fredrickson, 2019b); however, the design also provides good power to test our present hypothesis. Most couples were married (58.0%), engaged (11.0%), or dating exclusively (27.0%). All couples were cohabiting and had been together for more than 5 years (*M* = 5.41 year, *range* = 1–15 years). Participants’ ages ranged from 23 to 53 (*M =* 29.76, *SD* = 4.80). Most self-identified as White/Caucasian (77.9%) and the rest identified as African American (10.5%), East or South Asian (5.8%), American Indian or Pacific Islander (5.8%). In a separate question, 1.0% identified as Hispanic.

#### Procedure

Participants—both members of the couple—independently completed 28 nights of brief online reports, including their perceptions of partner responsiveness and reports of affectionate touch from that day, as part of a larger study (Algoe & Zhaoyang, 2016). A fuller description of the overall study procedure, which included an experimental manipulation at the first lab visit, where couples were randomly assigned to either express gratitude or self-disclose to their partner regularly over the course of the next month, and another lab visit after this 28-day period, can be found in Algoe and Zhaoyang (2016). Critically, the hypotheses in this study are about daily associations between variables, and we had no reason to predict that the in-lab manipulation would further influence the strength of those associations, so we do not take condition into account other than to use it as a control variable in analyses and to conduct an exploratory test of whether condition moderates effects (results are reported in the Online Supplemental material).

At the end of the first lab session, participants received instructions about what to expect in the coming weeks of the study, including an emphasis on the value of the nightly reports. Three couples did not send in any nightly questionnaires and were not included in the analyses. After excluding these non-compliant couples, the remaining participants completed 77.9% of the nightly questionnaires in the 28-day study (2,181 out of a possible 2,800 total reports), together achieving a sensitivity to detect a small effect|*ρ*| > .06 at power = 80% assuming no interdependence among data.

##### Daily measures

*Perceived Partner Responsiveness*. Participants reported on their partner’s responsiveness that day with one item (“I felt that my partner responded to my needs/wishes”) on a 5-point scale with anchors 1 (*very little or not at all*) to 5 (*very much*), *M* = 4.08 (*SD* at the day, individual, and couple level are 0.75, 0.43, and 0.34, respectively). Across the submitted 2181 nightly reports, 36 responses for this item were missing, or a 98.35% completion rate.

*Affectionate Touch*. Participants reported on three items about their affectionate touch in the past 24 hr with their partner, selected from the general affectionate touch measure reported in the IDA (Light et al., 2005; fewer were used for brevity in the nightly report). Specifically, we asked how often (0 = *not in the last 24 hr*, 1 = *once in the last 24 hr*, 2 = *several times in the last 24 hr*, 3 = *5 or more times in the last 24 hr*) they held hands; gave each other neck rubs, back massages or any other warm touching activities; and gave hugs lasting for more than a few seconds. These three items were averaged together to assess touch each day. Twenty responses to this item were missing, leaving 99.08% compliance.

*Sexual Intercourse*. Participants reported how often they had engaged in sexual intercourse that day, “including any actions that you, as a couple, consider sex.” The single item was measured on the same scale the affectionate touch items (0 *= not in the last 24 hr* to 3 = *5 or more times in the last 24 hr*). Thirty-two responses to this item were missing (a 98.35% compliance rate).

*Relationship Satisfaction*. Daily relationship satisfaction was measured on a scale from 1 (*terrible*) to 9 (*terrific*) with one item, “today our relationship was” (*M =* 7.6, *SD =* 1.19). Responses were missing from 19 nights (99.13% compliance rate).

### Results and Discussion

#### Conceptual models and data analysis plan

We tested our two hypotheses in two-level cross-classified models with random intercepts and fixed slopes of day-level predictors, in which individual partners were nested within couples and days were crossed to account for each partner completing a daily report (Kenny et al., 2006). We used a diagonal residual matrix, as in Study 1, to assume indistinguishability within the dyad. In the full results presented in Table 2, we parse daily variance into between-person effects and within-day deviations (Bolger & Laurenceau, 2013; Raudenbush et al., 2004).

**Table 2.** Test of Hypothesis 1 and 2 Using Disaggregated Within- and Between-Person Variance.

Predictor	*b*	*SE*	*df*	*t*	95% CI	
					Low	High
Hypothesis 1: perceived partner responsiveness → same day affectionate touch						
Within-person deviations of perceived partner responsiveness	0.16	0.02	1,682.29	9.81***	0.13	0.19
Prior day affectionate touch	0.38	0.02	1,712.79	17.92***	0.34	0.42
Between-individual differences in perceived partner responsiveness	0.16	0.06	71.78	2.75**	0.05	0.28
Experimental condition	−0.02	0.09	39.13	−0.22	−0.19	0.15
Hypothesis 2: affectionate touch → next day perceptions of touch-giver’s responsiveness						
Within-person deviations of affectionate touch	0.07	0.04	1,586.41	1.98*	0.00	0.14
Present day perceptions of touch-giver’s responsiveness	0.14	0.02	1,639.18	5.79***	0. 10	0.19
Between-individual differences in affectionate touch	0.11	0.10	76.02	1.09	−0.09	0.32
Experimental condition	0.04	0.12	40.45	0.36	−0.20	0.29

*Note.* CI = confidence interval; SE = standard error.

* *p* < .05. **p < .01. ***p < .001.

The first model tests Hypothesis 1, the theorized first step in the interpersonal process from Figure 1. Specifically, we tested for the effects of within- and between-person variability of perceived partner responsiveness on affectionate touch, by centering perceived partner responsiveness within individuals and predicting affectionate touch using both individual deviations in perceived partner responsiveness and the individual mean perceived partner responsiveness used for the centering. The means address the between-subjects effects, whereas the deviation term sets up a more conservative test of our theorized association from increases in perceived responsiveness to increases in affectionate touch. The second model tests Hypothesis 2, the theorized second step in the interpersonal process from Figure 1. Specifically, we tested for within- and between-person effects of daily affectionate touch on the *partner’s* perceptions of the touch-giver’s responsiveness the next day, using mean-centered affectionate touch to assess day-level changes and between-individual effects, and controlling for partner’s prior day perceptions of touch-giver’s responsiveness.

All models control for experimental condition (0 = active control; 1 = expressed gratitude). Additional models controlling for sexual intercourse produced consistent results and are reported in the Online Supplemental material. We also tested relationship satisfaction as an alternative explanation, running all models again while controlling for it. Finally, we ran ancillary analyses to explore the possible temporal sequence of Hypothesis 1 and Hypothesis 2; we ran a model using present day perceptions of partner responsiveness to predict affectionate touch the *next* day (i.e., prospective Hypothesis 1) and we conducted a test of present day affectionate touch predicting *same day* perceptions of touch-giver’s responsiveness (i.e., concurrent Hypothesis 2).

#### Hypothesis 1: Perceived partner responsiveness predicting same day affectionate touch

The results presented in the upper panel of Table 2 demonstrate that a tendency to perceive a partner as responsive (i.e., the average across days) accounts for a significant portion of the variance in change in affectionate touch from the prior day. Furthermore, even accounting for this general association, the results showed significant within-individual variation of perceived partner responsiveness predicting affectionate touch. Specifically, on any given day, participants who perceived their partner as more responsive than their average daily perception reported greater affectionate touch of the partner on those days, even when controlling for their own prior-day affectionate touch of the partner and between-individual mean-level differences in perceived responsiveness.

#### Hypothesis 2: Affectionate touch predicting partner’s perception of touch-giver’s responsiveness next day

Moving to the second hypothesis, and second step in a theoretical interpersonal process, the results presented in the lower panel of Table 2 support Hypothesis 2. Specifically, one’s average affectionate touch toward the partner across days significantly predicted the *partner* perceiving the touch-giver as more responsive the next day (i.e., at the between-person level), even when controlling for the partner’s perception of the touch-giver’s responsiveness on the present day. Critically, we also found significant associations with the within-individual daily deviations, such that on days where one’s affectionate touch was higher than their own average, the partner reported greater perceptions of the touch-giver’s responsiveness the next day, controlling for the partner’s present day perceptions of the touch-giver’s responsiveness and between-individual differences in affectionate touch.

#### Analyses of alternative hypotheses

Although we have demonstrated the two-step within-person associations between perceived partner responsiveness and affectionate touch day-to-day, it is possible that another daily situational variable, such as daily relationship satisfaction, is driving these effects. For Hypothesis 1, within-person fluctuations of perceived partner responsiveness continued to predict affectionate touch when present day’s relationship satisfaction was added to the model, even taking into account the between-person effect, which was no longer significant. For Hypothesis 2, after controlling for the relationship satisfaction of the *touch provider* on the day they provided touch, affectionate touch no longer predicted the touch receiver’s perceptions of the touch-provider’s responsiveness the next day. When controlling for *recipient’s* relationship satisfaction on the day they reported perceiving responsiveness, the within-person effect of participant’s reported affectionate touch (prior day) on recipient perceptions of participant responsiveness remained significant. Due to space constraints, full results for analyses can be found in the Online Supplemental material.

#### Ancillary analyses: Perceived partner responsiveness predicting next day affectionate touch

Hypothesis 1 tests theoretically ephemeral associations between perceived responsiveness and affectionate touch, so we focused on analyses within day (controlling for prior day touch); however, for exploratory purposes, we included lagged-day analysis of Hypothesis 1: perceived partner responsiveness predicting affectionate touch the *next day*, controlling for affectionate touch the present day. As in the original test, on average, greater perceptions of partner responsiveness (i.e., the between-subjects test) were associated with greater affectionate touch the next day, *b* = 0.16, *SE* = 0.06, *df* = 69.30, *t* = 2.66, *p* = .01, 95% CI = [0.04, 0.27], when controlling for present day affectionate touch, *b* = 0.40, *SE* = 0.02, *df* = 1,646.00, *t* = 18.25, *p* <.001, 95% CI = [0.36, 0.45], and experimental condition, *b* = −0.03, *SE* = 0.08, *df* = 38.45, *t* = −0.31, *p* = .76, 95% CI = [−0.19, 0.14]. However, the tests revealed no evidence of a within-person lagged effect in this model, *b* = 0.01, *SE* = 0.02, *df* = 1,699.37, *t* = 0.60, *p* = .55, 95% CI = [−0.02, 0.04]. See the Online Supplemental material for model and results controlling for present day relationship satisfaction.^7^

## Discussion

Despite a wealth of literature on the benefits of touch receipt (Jakubiak & Feeney, 2017), the extant literature reveals a gap regarding the potential situational precursors to providing affectionate touch. The current research presented some of the first empirical evidence related to this question, focusing on ecologically rich evidence from people in romantic couples. We proposed that perceiving a partner as responsive, a putative marker of psychological intimacy, would promote behavioral intimacy as measured by affectionate touch toward that partner. Our results, which marshal evidence from four distinct methodological approaches, are consistent with this hypothesis and open the door to future work on affectionate touch a behavioral instantiation of intimacy.

Specifically, an initial Integrative Data Analysis (IDA, Curran & Hussong, 2009) with four dyadic samples (*N* = 412 couples) documented a small positive association between greater general perceptions of the partner as responsive and greater frequency of self-reported affectionate touch of that partner over the prior month. Studies 1 and 2 focused on observations of affectionate touch behavior as it naturally arose between couple members. In Study 1, general perceptions of partner responsiveness at study entry forecasted unprompted reports of affectionate touch in subsequent daily descriptions of notable interactions with the partner. In Study 2, perceptions of partner responsiveness in one laboratory situation forecasted naturally occurring affectionate touch toward the partner and kissing when hanging out on a couch in a subsequent situation. Study 1 and 2 findings held when controlling for powerful individual differences in relational orientation, as well as relationship satisfaction (Study 2). Study 3 capitalized on the strength of within-person daily fluctuations across 28 days to show that the more a partner was perceived as responsive the more the participant affectionately touched that partner the same day, controlling for affectionate touch the day before. Critically, these effects could not be explained by daily fluctuations in the participant’s relationship satisfaction. Finally, though the focus has been on the touch-giver, in an extension of our theorizing about how this instantiation of the interpersonal intimacy process might unfold between people, related analyses documented that increases in affectionate touch on one day (relative to one’s average) predicted positive changes in the *partner’s* perception of the touch-giver’s responsiveness the following day. Together, these findings have implications for the literature on affectionate touch, bringing novel evidence to bear on one situational precursor to affectionate touch. They also contribute evidence to the interpersonal process of intimacy.

### Everyday Affectionate Touch

Affectionate touch is a normative behavior within close relationships. Our data show that touch is pervasive in the everyday moments between couples. Touch happens when sitting with a partner on a couch in the laboratory (Study 2) and over the course of a normal day (Studies 1 and 3). The use of natural behavioral data in Studies 1 and 2, including, respectively, coded affectionate touch in daily interactions with a partner and coded real-time affectionate touch, such as face caresses, light tickles, and kisses, provide a meaningful contribution to literature on affectionate touch. First, the observed behavior reiterates that affectionate touch is spontaneously demonstrated within close relationships and naturally occurring in everyday life. As an intimate gesture itself (Floyd, 2006), touch injects these seemingly small moments with feelings of intimacy that help facilitate high-quality connections. Although affectionate touch has been used as an experimental manipulation (Jakubiak & Feeney, 2016, 2019a) or as part of a functional magnetic resonance imaging (fMRI) study (Coan et al., 2006), self-reported in everyday life (e.g., romantic couples, Debrot et al., 2013; middle-aged women, Burleson et al., 2007), and categorized (e.g., from self-reports, Jones & Yarbrough, 1985), this research is some of the first to use behaviorally coded affectionate touch as the outcome of interest.

Moreover, we believe it is one of the first to capture this behavioral intimacy as it emerges naturally between romantic couples. The affectionate touch behavioral codes, which were validated against participant’s self-reports of affectionate touch, are easy to train on and replicate, and may be useful for other labs to apply to future work. Notably, a recent study by Jakubiak and Feeney (2019b) had objective raters code for touch (i.e., categorizing affectionate, casual, and playful touch) demonstrated in videorecorded stressor discussions between couple members. While those researchers used touch as a predictor rather than an outcome, it might be interesting to examine whether perceived responsiveness predicts categorically different types of touch in various dyadic interactions.

Critically, the current research focused intentionally on non-sexual affectionate touch (e.g., touch in the lab in Study 2; holding hands, neck rubs, hugs in Study 3), and analyses in all studies controlled for sexual intercourse, to indirectly rule out affectionate touch that was motivated by communicating feelings of attraction or sexual interest (Jones & Yarbrough, 1985). We have demonstrated that the study of everyday affectionate touch can be independent from the study of everyday sexual intercourse, and specifically, that perceived responsiveness can independently predict everyday affectionate touch apart from the likelihood of having sexual intercourse (a useful distinction from work showing responsiveness promotes sexual desire; Birnbaum, Reis et al., 2016). However, evidence from Study 1 and Study 3 highlights that sexual intercourse is also predictive of affectionate touch behavior. This links well with work on the importance of affectionate behavior following sexual intercourse for sexual and relationship satisfaction (Hughes & Kruger, 2011; Muise et al., 2014; van Anders et al., 2013). Of interest, we did *not* observe correlations between ecologically valid affectionate touch and important individual differences like attachment (Study 1), communal strength, or relationship satisfaction (Study 2). Collectively, these findings raise intriguing questions about the most important precursors to affectionate touch behavior that we look forward to examining in future work.

### Situational Precursors to Affectionate Touch

Beyond documenting that affectionate touch happens in everyday life, our central question was about what situations might give rise to it. While previous work has examined the cultural or individual factors that may increase the likelihood of preferring or receiving touch, the situational antecedents of touch have been overlooked in the literature. Here, we rested on the threads of evidence already present within theory on the interpersonal process model of intimacy as well as within the affectionate touch literature to make our prediction.

Although all evidence is correlational, our findings are consistent with the theorized direction of effects, such that perceiving a partner as responsive—an instantiation of psychological intimacy—is associated with greater subsequent affectionate touch—an instantiation of behavioral intimacy. As our strongest evidence, we see prospective associations in the laboratory that cannot be explained by an individual’s communal orientation (Study 2), and the within-person analyses documenting greater perceptions of partner responsiveness from one’s own average perception being associated with positive change in affectionate touch on the same day (i.e., controlling for prior day) and that cannot be explained by concomitant deviations in daily relationship satisfaction (Study 3). At the same time, the breadth of situations we cover—in terms of potential for responsive behavior as well as everyday situations in which touch arises—helps add strength to our argument that these processes likely exist in everyday life and merit researchers’ attention. Of course, we also cannot and do not rule out the potential reverse causal pathway in Studies 1 and 3. In Study 1, because we were effectively assessing general perceptions of responsiveness and general tendencies to enact touch behavior in daily life, we do not have evidence about which came first; furthermore, in Study 3, because perceived responsiveness and affectionate touch were measured simultaneously at the end of the day, we cannot determine order. Nonetheless, taken together the present package of tests fills a gap in the affectionate touch literature by critically examining a viable situational precursor to affectionate touch.

### Affectionate Touch as Part of an Intimate Interpersonal Process

Prior work has documented the role of perceived partner responsiveness in facilitating other relationship behaviors (e.g., sexual desire, Birnbaum, Reis, et al., 2016; approach behavior, Birnbaum, Mizrahi, et al., 2016; emotion expression, Ruan et al., 2020). Our findings add affectionate touch to the list of possible behavioral outcomes of perceiving a partner as responsive. Critically, affectionate touch, a behavior which manifests spontaneously in small moments throughout the day, is particularly important for relationship functioning (Debrot et al., 2013; Jakubiak & Feeney, 2017, 2019b) and is a behavior that is perceived as intimate (Debrot et al., 2012, 2013; Guerrero & Andersen, 1991; Thayer, 1986). Given this growing body of evidence, it is an opportune moment within the field to pick up on Reis and Shaver’s (1988) original discussion of affectionate touch within the interpersonal intimacy process, that given: “the prominence of touch, gazing, etc., in adult romantic interactions, and the importance of nonverbal factors in communicating openness, nonverbal components of intimacy are especially central in a process model meant to apply . . . across the lifespan” (p. 373).

Beyond our evidence related to the question of whether there is a within-person effect, such that one person’s perceptions of a partner’s responsiveness could make them reach out and touch their partner, we considered the subsequent theoretically plausible step, which was that there would be a cross-partner effect, such that the touch-giver’s partner would perceive them as more responsive. Indeed, our research provides initial evidence in support of the theoretical model suggesting affectionate touch is a mechanism at the heart of the interpersonal intimacy process. In Study 3, one’s own greater behavioral intimacy as measured by self-reported affectionate touch on a given day was associated with the partner’s greater perceptions of the touch-giver’s responsiveness that same day and the next day. The within-person analyses lend strong support of our theorizing at the state- or day-level while between-person effects suggest more trait-level processes. At the same time, controlling for relationship satisfaction called into question the robustness of the finding between days and alludes to a more fine-grained temporal sequence (i.e., contemporaneous) of this step in the interpersonal process.

We could find only two other studies that conduct similar tests as our second hypothesis (Debrot et al., 2012, 2013), reporting concurrent daily associations between “responsive touch” or “enacted responsiveness” and partner’s psychological intimacy, but with no support for lagged effects as we find in Study 3. In that research, however, both the independent and dependent variables were operationalized in different ways than in our present investigation, suggesting the need for additional research to fully test the conceptual model of interest. Altogether, these data support the interpersonal process model of intimacy put forth by Reis and Shaver (1988), specifically suggesting a process of mutual cyclical growth. We hope this potentiates mechanistic tests of the interpersonal intimacy process in future work.

### Limitations and Future Directions

Although we conceptually replicated our finding across four well-powered tests, we acknowledge some important limitations to this work. The data presented are correlational in nature. To determine the true causal nature of the relationship between perceived responsiveness and affectionate touch, we need experimental evidence that greater perceived responsiveness (compared with lower) leads to greater affectionate touch. Unfortunately, affectionate touch is not a behavior that can be easily observed via online experiments, unlike some other social behaviors (e.g., generosity; Haley & Fessler, 2005), so creativity and additional resources will be needed to measure situational touch induced from manipulated perceptions of partner responsiveness.^8^ Furthermore, this study focused on romantic couples in the United States. Although work suggests touch may operate differently based on relationship type (Suvilehto et al., 2019), we have no reason to predict that this theorized intimate process should function differently, albeit perhaps of a different magnitude, in non-romantic relationships. However, the possible moderation of our findings by relationship type or other contextual factors, such as culture, remains an empirical question.

We also note that while touch is important in a wide array of situations (e.g., making up after conflict, consoling someone suffering), we do not have strong evidence about whether this particular process we propose—perceiving a partner as responsive causing one to reach out and touch that person—happens in situations that are negative in valence; another possibility is that the effect size is different depending on the valence of the situation. Specifically, Study 2 is a situation that is positive in valence. Studies 2 and 3 likely include touch in situations that are negative in valence, but we do not have direct evidence related to that question. Yet, it is a theoretically interesting one, in part because some positive interpersonal processes—that is, social interactions fueled by positive valence (Algoe, 2019)—have had more robust relationship-promoting effects than beneficial social interactions involving negative emotions in prior research (e.g., Algoe et al., 2013; Gable et al., 2012).

Beyond a scholarly contribution, the present research might have useful translational consequences. It is well documented that touch—especially affectionate touch—seems to provide physiological benefits (Ditzen et al., 2008; Holt-Lunstad et al., 2008; Light et al., 2005). As such, increasing opportunities for safe affectionate touch among couples is an important area for consideration in the research community. The current evidence suggests that finding ways to *promote perceived responsiveness* within romantic relationships can make way for more affectionate touch within them. This is true for both members of the dyad, as the interpersonal process may theoretically jump start a mutual process that translates to cumulative physiological benefit by way of increases in affectionate touch.

## Conclusion

Our study documents the normative, spontaneous, myriad ways affectionate touch occurs in couple’s everyday life and highlights the value of employing behavioral data in the study of touch. Reis and Shaver (1988) argue that fundamental intimacy-building behaviors are present during development; affectionate touch is a keystone behavior in infant–caregiver interactions (Ferber et al., 2008; Hertenstein et al., 2006) and remains important for social bonds throughout the lifespan (Burleson et al., 2007; Guerrero & Andersen, 1991). The present research provides the first tests of a theorized situational precursor to affectionate touch and begins to unpack whether perceived partner responsiveness, as a gesture of psychological intimacy, undergoes a translation of motivation to promote behavioral intimacy as measured by affectionate touch. If so, this would implicate affectionate touch as a theoretical mechanism for relational, psychological, and physical benefits of perceived partner responsiveness (e.g., Caprariello & Reis, 2011; Maisel & Gable, 2009; Pansera & La Guardia, 2012; Reis & Clark, 2013; Selcuk et al., 2016, 2017; Slatcher et al., 2015; Stanton et al., 2019). Altogether, the current findings offer a substantial advance in the literature on affectionate touch and provide meaningful considerations for its role in the intimacy process as it unfolds in couples’ everyday lives.

## Supplemental Material

sj-docx-1-psp-10.1177_0146167221993349 – Supplemental material for Perceived Partner Responsiveness Forecasts Behavioral Intimacy as Measured by Affectionate Touch Click here for additional data file. Supplemental material, sj-docx-1-psp-10.1177_0146167221993349 for Perceived Partner Responsiveness Forecasts Behavioral Intimacy as Measured by Affectionate Touch by Tatum A. Jolink, Yen-Ping Chang and Sara B. Algoe in Personality and Social Psychology Bulletin

sj-docx-2-psp-10.1177_0146167221993349 – Supplemental material for Perceived Partner Responsiveness Forecasts Behavioral Intimacy as Measured by Affectionate Touch Click here for additional data file. Supplemental material, sj-docx-2-psp-10.1177_0146167221993349 for Perceived Partner Responsiveness Forecasts Behavioral Intimacy as Measured by Affectionate Touch by Tatum A. Jolink, Yen-Ping Chang and Sara B. Algoe in Personality and Social Psychology Bulletin

## Notes

1. Since first proposed in the interpersonal process model of intimacy, perceived partner responsiveness has consistently been theorized as an instantiation of intimacy. Behavior, like affectionate touch, being an instantiation of intimacy is less well established, but very consistent with evidence.

2. Assuming independence, or purposefully overestimating independence and randomness to their maximum, means underestimating the relative sizes of effects and, therefore, estimating the data to the conservative lower bound of their power. We use this approach throughout the article.

3. Please see Studies 1 to 3 for more information about each individual sample (Sample A, B and C, respectively), with descriptive information about a fourth sample, Sample D, reported in the Online Supplemental material.

4. Due to how touch is often described in first person plural (e.g., “we cuddled on the couch,” “we kissed”), we chose to include all mentions of touch. This means reports where the participant initiated, reports where the partner-initiated, and reports where initiation was ambiguous were all counted.

5. We note that some suggest using an unstructural matrix (see syntax in Bolger & Laurenceau, 2013). This means the two partners are still distinguished, statistically, in a cross-classified model, whereas using the diagonal matrix in the cross-classified model allows for the assumption that they are indistinguishable in their effects.

6. In one video, it was ambiguous as to whether the Target or Expresser initiated the kiss, but this observation was retained to keep as much data as possible. Results do not change if video was recoded as 0.

7. For those interested, we also conducted an exploratory model of Hypothesis 2, using present day affectionate touch to predict partner’s perceptions of touch-giver’s responsiveness the *same day*, controlling for that perception the prior day. Full results, including those controlling for relationship satisfaction, are in the Online Supplemental material.

8. We acknowledge that two studies manipulate thoughts about partner responsiveness; however, they do not document an influence on *perceptions* of partner responsiveness, our mechanism of interest (Reis et al., 2018; Ruan et al., 2020) so more work is needed in this domain.
