# Supplementary material for: Perceived Partner Responsiveness Forecasts Behavioral Intimacy as Measured by Affectionate Touch
Source: Pers Soc Psychol Bull. 2021 Mar 19;48(2):203–21. doi: 10.1177/0146167221993349 (PMC8801651; doi:10.1177/0146167221993349)
Supplement: sj-docx-1-psp-10.1177_0146167221993349 – Supplemental material for Perceived Partner Responsiveness Forecasts Behavioral Intimacy as Measured by Affectionate Touch [file sj-docx-1-psp-10.1177_0146167221993349.docx]

**Preliminary Study**

**Integrative Data Analysis: Testing the Basic Concurrent Association across Four Samples**

**Perceived Partner Responsiveness**

The extent to which participants feel their partner is responsive to them in general was assessed with an 18-item scale assessing understanding, validation, and caring (Reis, Crasta, Rogge, Maniaci, & Carmichael, 2017). Items include, “My partner understands me,” “My partner is responsive to my needs,” and “My partner sees the ‘real’ me” measured on a 7-point Likert scale ranging from 1 (*not at all true/never*) to 7 (*very true/true all of the time*). Sample D used the 12-item scale (Reis, Maniaci, Caprariello, Eastwick, & Finkel, 2011). For the IDA, we harmonized the scales of different data sources by standardizing them within their respective samples before merging them together. The average reliability across the four samples was acceptable, α =.94; α_min_ =.92 and α_max_ =.95.

**Full 18-item perceived partner responsiveness measure used in Samples A-C:**

Citation: Reis, H. T., Crasta, D., Rogge, R. D., Maniaci, M. R., & Carmichael, C. L. (2017). Perceived Partner Responsiveness Scale (PPRS) (Reis & Carmichael, 2006). *The sourcebook of listening research: Methodology and measures*, 516-521.

Instructions: The statements below reflect different aspects of your relationship. Please use the scale below to indicate how much each statement applies to your relationship.

| 1 | 2 | 3 | 4 | 5 | 6 | 7 |
| --- | --- | --- | --- | --- | --- | --- |
| Not at All True/Never True |  |  | Moderately True/True Half of the Time |  |  | Very True/True all of the Time |

1. My partner is an excellent judge of my character.
2. My partner sees the “real” me. _____
3. My partner sees the same virtues and faults in me as I see in myself.
4. My partner “gets the facts right” about me.
5. My partner esteems me, shortcomings and all.
6. My partner knows me well.
7. My partner values and respects the whole package that is the “real” me.
8. My partner usually seems to focus on the “best side” of me.
9. My partner is aware of what I am thinking and feeling. ____
10. My partner understands me.
11. My partner really listens to me. ____
12. My partner expresses liking and encouragement for me.
13. My partner seems interested in what I am thinking and feeling.
14. My partner seems interested in doing things with me.
15. My partner values my abilities and opinions.
16. My partner is on “the same wavelength” with me.
17. My partner respects me.
18. My partner is responsive to my needs.

**Full 12-item perceived partner responsiveness measure used in Sample D:**

Citation: Reis, H. T., Maniaci, M. R., Caprariello, P. A., Eastwick, P. W., & Finkel, E. J. (2011). Familiarity does indeed promote attraction in live interaction. *Journal of Personality and Social Psychology*, *101*(3), 557.

Instructions: The statements below reflect different aspects of your relationship. Please use the scale below to indicate how much each statement applies to your relationship.

| 1 | 2 | 3 | 4 | 5 | 6 | 7 |
| --- | --- | --- | --- | --- | --- | --- |
| Not at All True/Never True |  |  | Moderately True/True Half of the Time |  |  | Very True/True all of the Time |

1. My partner sees the “real” me.
2. My partner “gets the facts right” about me.
3. My partner esteems me, shortcomings and all.
4. My partner knows me well.
5. My partner values and respects the whole package that is the “real” me.
6. My partner understands me.
7. My partner really listens to me.
8. My partner expresses liking and encouragement for me.
9. My partner seems interested in what I am thinking and feeling.
10. My partner values my abilities and opinions.
11. My partner is on “the same wavelength” with me.
12. My partner is responsive to my needs.

**Affectionate Touch**

Participants reported on the frequency of physical affection with their partner in the past month (Light, Grewen, & Amico, 2005). The scale evaluates how frequently each behavior occurred in the past month from 0 (*never or almost never*) to 6 (*five or more times a day*). The items were: “how often do you hold hands with your spouse/partner?,” “how often do you sit close together or lie down close together with your spouse/partner while reading, watching TV or other leisurely activities?”, “how often do you give each other neck rubs, back massages or any other warm touching activities?”, “how often do you give your spouse/partner hugs lasting for more than a few seconds?”, “how often do you kiss your spouse/partner?”. We computed the mean of the five items as our indicator of affectionate touch. All scales were standardized within the sample before merging and the average reliability across the four samples was satisfactory, α =.81; α_min_ =.78 and α_max_ =.84.

**Sexual Intercourse**

An additional item, “how often do you and your partner have sex (intercourse)?,” which was measured on the same scale as the affectionate touch items, from 0 (*never or almost never*) to 6 (*five or more times a day*), was included to test as a potential covariate.

**Full affectionate touch measure, including sexual intercourse item:**

Citation: Light, K. C., Grewen, K. M., & Amico, J. A. (2005). More frequent partner hugs and higher oxytocin levels are linked to lower blood pressure and heart rate in premenopausal women. *Biological Psychology*, *69*(1), 5-21.

Instructions: For the following questionnaire, please indicate how often you do each activity below. Think of the last month when you have lived together and choose the answer choice that most closely reflects that month’s activities.

| 0 | 1 | 2 | 3 | 4 | 5 | 6 |
| --- | --- | --- | --- | --- | --- | --- |
| Neve or almost never | Less than once a week but at least once a month | About once a week | Several times a week | About once a day | Several times a day | Five or more times a day |

1. How often do you hold hands with your spouse/partner?
2. How often do you sit close together or lie down close together with your

spouse/partner while reading, watching TV or other leisurely activities?

1. How often do you give each other neck rubs, back massages or any other warm

touching activities?

1. How often do you give your spouse/partner hugs lasting for more than a few seconds?
2. How often do you kiss your spouse/partner?

**Sexual intercourse item, assessed using the same scale as affectionate touch items:**

1. How often do you and your partner have sex (intercourse)?

**Study 1: Prospective Link From General Perceptions of Responsiveness to Everyday Spontaneously-Reported Affectionate Touch**

**General Perceived Partner Responsiveness**

Participants completed the self-reported general perception of responsiveness from the initial lab session (described and included in the IDA on page 2, Sample B; α = .92)

**Coded Spontaneously-Reported Affectionate Behavior**

During a two-week daily diary, participants completed a novel nightly measure in which they briefly described the most notable interaction with their partner that day. The specific measure of interest from the nightly questionnaire was an open-ended response that participants gave to a prompt to briefly describe an interaction with their partner that “made the biggest impression today.” For this item, 380 reports out of the received 1860 (79.57% compliance) did not contain any content and thus were not coded for analysis.

As described above, the prompt participants received did not explicitly instruct them to write about affectionate touch. The actual events participants wrote about may have been minor or major, positive or negative, but critically, upon reading the whole corpus, we noticed that the interactions with the partner that made the biggest impression contained several instances of explicit mention of affectionate touch. Hence, we coded the text for the number of spontaneous reports affectionate touch. Two independent judges were trained to code the nightly data. After training, coders practiced by independently working through the same 150 responses to establish reliability (8% of the total nightly responses in the study). Then, the remaining 1710 responses were independently coded by each rater. Throughout, all coders were unaware of the hypothesis.

The coding instructions were modeled after the count code in Study 1 as well as the physical affection scale employed as part of the IDA (Light, Grewen, & Amico, 2005). Specifically, coders were instructed: “Count each unique instance of affectionate touch mentioned in the nightly event description. Affectionate touch is warm physical contact that communicates fondness and positive regard, as well as love and support. It can take many forms, but some instantiations are hugging, kissing, massaging, and cuddling, and some less obvious versions may be sitting close together or lying down close together doing leisure activities, sleeping/spending the night together or dancing. Further, based on context, there also may be euphemisms of affectionate touch – those count too.” For example, “[my partner] came by work and saw me real quick. its always nice to see her in the middle of the day and give her a hug and kiss” would have been coded as a 2. Notably, some participants mentioned desiring affectionate touch from their partner and not receiving it. Any mention of a touch “deficit” was not included in the count. In addition, some phrases seemed highly likely to include affectionate touch, so were included, despite the fact that they also may have included sexual activity (e.g., “get intimate”, “spent the night together”); because of this, the count code also included explicit mentions of “sexual intercourse.” Finally, in some responses we were unable to discern who initiated the touch (e.g., “All day there were many hugs and kisses. I bought breakfast and she cooked it just for me. We also watched a movie together hugged up on the sofa. That was the best hour and 43 mins of the day”), so we coded every mention of touch.

Coders noted anywhere from 0 to 5 instances of affectionate touch (ICC = .89). See Table 2 for an example of each code. For 4.2% of responses (N = 79) coders had different counts of affectionate touch and a third rater (the first author) arbitrated based on fidelity to the definition.

**Full affectionate touch coding instructions for self-report:**

Count each unique instance of affectionate touch mentioned in the nightly event description. Affectionate touch is warm physical contact that communicates fondness and positive regard, as well as love and support. It can take many forms, but some instantiations are hugging, kissing, massaging, and cuddling, and some less obvious versions may be sitting close together or lying down close together doing leisure activities, sleeping/spending the night together or dancing. Further, based on context, there also may be euphemisms of affectionate touch – those count too.

Note, some instances of touch may have occurred in the past day and others are reports of touch that haven’t happened yet or will in the future. For instance, in some reports, the participant mentions being promised something in the future or their partner alluding to touch from the past, present or future. Either counts toward the count.

Also, the nightly event descriptions are truncated. Some may cut off mid-sentence or before they’re done – this is a function of the survey platform used to collect the data. Just do your best when this happens.

After counting each unique instance of touch, put the total count in the column to the right of the nightly event descriptions, AT_count. If an entry has no instances of touch, make sure you code it as 0. Every entry should have a count, except the ones that are blank!

***Sexual Intercourse Coding***

Coders classified whether certain affectionate touch behaviors were present (coded as 1) or absent (0) in the description. Specifically, judges marked the presence/absence of sexual intercourse, as a potential control variable. Coder reliability was high (all ICCs were at least .85) and final determinations of discrepancies was determined by consensus among the six coders.

**Full sexual intercourse coding scheme instructions:**

| Behavioral codes | |
| --- | --- |
| 0 | absent |
| 1 | present |
| **Touch Behavior** | |
| Sex/get intimate/sexual activity | |

**Testing Alternative Explanations: Attachment**

**Full 35-item Experiences in Close Relationships scale:**

Citation: Brennan, K. A., Clark, C. L., & Shaver, P. R. (1998). Self-report measurement of adult attachment: An integrative overview. In J. A. Simpson & W. S. Rholes (Eds.), Attachment theory and close relationships (pp. 46–76). New York: Guilford.

Instructions: The following statements concern how you feel in romantic relationships. We are interested in how you generally experience relationships, not just in what is happening in your current relationship. Respond to each statement by indicating how much you agree or disagree with it. Write the number in the space provided, using the following rating scale.

| 1 | 2 | 3 | 4 | 5 | 6 | 7 |
| --- | --- | --- | --- | --- | --- | --- |
| Strongly Disagree |  |  | Neutral/Mixed |  |  | Strongly Agree |

1. I prefer not to show a partner how I feel deep down
2. I worry about being abandoned
3. I am very comfortable being close to romantic partners.
4. I worry a lot about my relationships.
5. Just when my partner starts to get close to me I find myself pulling away.
6. I worry that romantic partners won’t care about me as much as I care about them.
7. I get uncomfortable when a romantic partner wants to be very close
8. I worry a fair amount about losing my partner.
9. I don’t feel comfortable opening up to romantic partners.
10. I often wish that my partner’s feelings for me were as strong as my feelings for him/her.
11. I want to get close to my partner, but I keep pulling back.
12. I often want to merge completely with romantic partners, and this sometimes scares
     them away.
13. I am nervous when partners get too close to me.
14. I worry about being alone.
15. I feel comfortable sharing my private thoughts and feelings with my partners
16. My desire to be very close sometimes scares people away.
17. I try to avoid getting too close to my partner.
18. I need a lot of reassurance that I am loved by my partner.
19. I find it relatively easy to get close to my partner
20. Sometimes I feel that I force my partners to show more feeling, more commitment.
21. I find it difficult to allow myself to depend on romantic partners.
22. I do not often worry about being abandoned.
23. I prefer not to be too close to romantic partners
24. If I can’t get my partner to show interest in me, I get upset or angry
25. I tell my partner just about everything.
26. I find that my partner(s) don’t want to get as close as I would like.
27. I usually discuss my problems and concerns with my partner.
28. When I’m not involved in a relationship, I feel somewhat anxious and insecure.
29. I feel comfortable depending on romantic partners
30. I get frustrated when my partner is not around as much as I would like.
31. I don’t mind asking romantic partners for comfort, advice, or help.
32. It helps to turn to my romantic partner in times of need.
33. When romantic partners disapprove of me, I feel really bad about myself.
34. I turn to my partner for many things, including comfort and reassurance.
35. I resent it when my partner spends time away from me.

**Study 2: Prospective Link From In-Situ Perceptions of Responsiveness to Subsequent Affectionate Touch in Lab**

**Experimental Manipulation**

Instructions for selecting the event and having the expressed gratitude conversation followed the standard paradigm (Citation Blinded), with the caveat that just prior to the conversation, in a different room across the lab suite, the Expresser had been privately given additional guidance about *how* to express, without the Target’s knowledge. That experimental manipulation, the fact that it had no effect on the outcome of interest – Target’s perceived partner responsiveness – and why that may have been, are all extensively documented in a prior publication (Citation Blinded). Here, our interest was in whether perceptions of partner responsiveness (which were not affected by the manipulation) forecasted a subsequent behavior, so we collapse across condition in analyses. Nevertheless, because the instructions may have influenced the Expresser’s behavior in the interaction, we control for condition in analyses.

Specifically, participants were randomly assigned to either focus on “the praiseworthiness of your partner’s actions” or focus on “the positive outcome you personally received in the situation” when expressing gratitude to their partner in an upcoming interaction. The manipulation was simple, but tight.

**Perceived Expresser Responsiveness After Expressed Gratitude Conversation**

Immediately following the conversation, Targets rated their perceptions of the Expresser’s responsiveness during the previous interaction using a 10-item situational measure of responsiveness (Gable, Gonzaga, & Strachman, 2006). The scale ranged from 0 (*not true at all/never true*) to 6 (*very true/true all of the time*), with 3 representing (*moderately true/true all of the time*), and higher numbers representing greater perceived partner responsiveness during the gratitude expression task. The reliability the Target’s rating of these items was satisfactory (α = .94).

**Full 10-item scale of perceptions of partner responsiveness during the interaction:**

Citation: Gable, S. L., Gonzaga, G. C., & Strachman, A. (2006). Will you be there for me when things go right? Supportive responses to positive event disclosures. *Journal of Personality and Social Psychology*, *91*(5), 904.

Instructions: Please use the scale below to indicate how much of each of the following statement applies to the previous interaction here in the laboratory.

| 0 | 1 | 2 | 3 | 4 | 5 | 6 |
| --- | --- | --- | --- | --- | --- | --- |
| Not at All True/Never True |  |  | Moderately True/True Half of the Time |  |  | Very True/True all of the Time |

1. My partner saw the “real” me.
2. My partner “got the facts right” about me.
3. My partner focused on the “best side” of me.
4. My partner was aware of what I was thinking and feeling.
5. My partner understood me.
6. My partner really listened to me.
7. My partner expressed liking and encouragement for me.
8. My partner valued my abilities and opinions.
9. My partner respected me.
10. My partner was responsive to my needs.

**Behavioral Coding: Affectionate Touch**

Using the videorecorded private leisure time that occurred later in the session, two trained coders separately watched the five-minute video without sound and coded it for the Target’s affectionate touch of the Expresser. The coders received thorough training that included two weeks of practice coding (15 videos) with regularly scheduled meetings to discuss and “recalibrate” codes. After reliable scores were reached, the two coders independently coded the 125 videos for the focal behavior: affectionate touch. Throughout the behavioral coding, coders were kept unaware of the hypothesis.

Affectionate touch was defined as “warm physical contact that communicates fondness and positive regard, as well as love and support” that “can take many forms but some instantiations are hugging, kissing, stroking, and cuddling” and be done with “the hand, the whole body, or any other part such as head, foot, and lips.” Affectionate touch did not include functional touches, e.g., accidentally brushing hands when turning the pages of the magazine, or aggressive touches, e.g., intentionally hurtful, non-playful pinches or pokes. The judges coded affectionate touch in two ways.

First, two coders documented each unique instance of when the Target touched the Expresser affectionately (ICC = .88), which created a count variable of the number of enacted affectionate touches from Target to Expresser during the five minutes. For example, if the Target 1) leaned into the crook of the Expresser’s open arm, during which s/he 2) patted the Expresser’s leg, 3) nudged the Expresser with his/her foot and 4) patted the Expresser’s leg again, the coders would code the Target as having demonstrated affectionate touch 4 unique times during that set of behavior. Coders observed anywhere from 0 to 32 instances of affectionate touch within the 5-minute period. A difference score was computed between the two coders. For any videos with a difference score of 3 or less, the two coder’s scores were averaged together. For 8% of videos (N = 10), coders reported a difference score of more than 3 and a third rater (the first author) arbitrated based on fidelity to the training instructions.

Second, coders also provided an overall affectionate touch *rating* for each video; because results are similar, in the interest of space these methods and results are described in the OSM.

**Full affectionate touch coding scheme instructions:**

Definition: Affectionate touch is warm physical contact that communicates fondness and positive regard, as well as love and support. Can take many forms but some instantiations are hugging, kissing, stroking, and cuddling.

TO CODE IN NOLDUS

Video Codes

Code for anytime newcomer displays any kind of affectionate touch toward the sitter. Touch could be with the hand, the whole body, or any other part such as head, foot, and lips.

**Count every instance of affectionate touch from newcomer to sitter.**

For example, if the sitter puts his/her arm around the newcomer that would not count as an instance. But if the newcomer then leans into the sitter’s open arm, that would could as one instance.

Sticky instances:

There will be variation in touch length. Some touches – e.g. cuddling – may last minutes. Others – a poke or kiss – may be brief. Cuddling (for 1.5 minutes let’s say) and a kiss on the cheek would both count as one touch each.

However, if during the cuddle the newcomer does other, distinct touch behaviors, each instance would count (like nudges the sitter with his/her foot and rubs arm = 2 touches).

Stroking with thumb or hand: the “stroking” is one instance, not each “back-and-forth” of the thumb or hand. When contact is made and sustained, that’s one touch.

Disclaimer: in broader context of life, some of these behaviors – poking and pinches – aren’t affectionate. But should be able to tell. Chances are those kinds of behaviors are going to be affectionate. Have a playfulness to them. In this context, chances they are negative is going to be super small. However, we *wouldn’t* want to count hurtful/aggressive touches. Look back at the definition if you’re unsure.

Also, don’t count “functional” touches – like brushing hands when turning page of magazine

Video Ratings (don’t need to memorize this since it’s in the survey. Just adding it here so you can also look back at conceptual definition if need to)

Answer the following one question after you have coded the entire video. Think of the *entire* interaction – not one section of the video or one behavioral instance – when you answer for the *newcomer*: (1=none at all, 5=a great deal)

1. How much affectionate touch did the newcomer demonstrate? I.e. warm physical contact that communicates fondness, positive regard, love and support.

**Behavioral Coding: Kissing**

Separately, videos had been coded by a prior team for whether the couples kissed, and this included kisses that had been initiated by the Expresser (ICC = .97). Most of the kisses were brief and although many were kisses on the partner’s lips, some were kisses on top of the partner’s head, on the partner’s shoulder, or on a hand. For the present investigation, the first author (unaware of participants’ ratings of responsiveness) eliminated instances where the only kiss within the video was obviously initiated by the Expresser, resulting in a code documenting any evidence of target-initiated kissing within a video as indicating the presence (1) of this strong signal of behavioral intimacy; videos that did not include a kiss from the target to the expresser were coded as (0). These criteria eliminated six videos where the only kiss was initiated by the expresser (coded as 0), leaving a total of 27 videos that included a kiss initiated by the Target. ^[[1]](#footnote-1)^

**Behavioral Coding: Expresser Engagement**

To address the possibility that the proposed effect on Target’s affectionate touch could be accounted for by the Expresser’s behavior in the new situation, rather than the Target’s perception of the Expresser’s responsiveness in the prior situation, four coders rated how interested and engaged the Expresser was toward the Target from the time the Target entered the primary lab room until they sat down on the couch. Expresser engagement was rated at the following levels: 1=disinterested (i.e., unaware or disinterested in partner’s return); 2=acknowledgement (i.e., looks up upon partner’s return); 3=interested and welcoming (i.e., smiles or has look of anticipation upon partner’s return); 4=excited and very welcoming (i.e., high levels of engagement upon partner’s return, such as continued positive facial expressions, making physical room on the couch, displaying welcoming arm gestures), *M* = 2.50, *SD* = 0.89. ICC for the four coders was 0.94. This variable is used as a control variable in analyses.

**Full expresser interest coding scheme instructions:**

What is the level of engagement and interest demonstrated by Expresser from the moment Target enters the door to the moment Target takes a seat (otherwise known as the welcoming scene). Pay attention to only the nonverbal behavior of Expresser. Try to determine how the Expresser is processing their partner’s return by looking at their body language. Do they seem like they were anticipating Target’s arrival or do they seem to be engrossed in the magazine?

· Rate Expresser’s level of interest by with a 1, 2, 3, or 4:

1 Disinterested – mark 1 if the Expresser seems to be unaware or disinterested in their partner’s return. If Expresser continues to look down as Target enters the room, walks over, and takes a seat, this is considered to be a “1”.

2 Acknowledgement- mark 2 if Expresser acknowledges Target’s return by looking up at Target. You should mark 2 if Expresser shows that they are at least aware of Target’s presence, but not particularly excited or interested. For example: if Expresser looks up as Target enters the room but then looks back down at the magazine as Target walks over.

3 Interested and Welcoming – mark 3 if Expresser acknowledges Target’s welcomed return by smiling; having a look of anticipation as participant walks over; if Expresser continues to gaze at Target as they walk over and take a seat, etc. Pay attention to how engaged Expresser seems to be with Target as Target approaches the couch.

4 Excited and very welcoming – mark 4 if Expresser really seems to be excited that Target has returned. High levels of engagement and excited can be demonstrated by continuous positive facial expressions (such as smiling and raised eyebrows) as Target walks over to the couch; physically making room on couch for Target’s arrival; displaying welcoming arm gestures (such as putting one arm on top of couch for example), putting magazine down and/or off to the side

**Testing Alternative Explanations: Communal Strength and Relationship Satisfaction**

**Full 10-item Communal Strength scale:**

Citation: Mills, J., Clark, M. S., Ford, T. E., & Johnson, M. (2004). Measurement of communal strength. *Personal relationships*, *11*(2), 213-230.

Instructions: Keeping in mind the specific person, answer the following questions. As you answer each question, fill in the person’s initials in the blank. Circle one answer for each question on the scale from *0: not at all to 10: extremely* before going on to the next question. Your answers will remain confidential.

| 1 | 2 | 3 | 4 | 5 | 6 | 7 | 8 | 9 | 10 |
| --- | --- | --- | --- | --- | --- | --- | --- | --- | --- |
| Not at All |  |  |  |  |  |  |  |  | Extremely |

1. How far would you be willing to go visit ____?
2. How happy do you feel when doing something that helps ___?
3. How large a benefit would you be likely to give ___?
4. How large a cost would you incur to meet a need of ___?
5. How readily can you put the needs of ___ out of your thoughts?
6. How high a priority for you is meeting the needs of ___?
7. How reluctant would you be to sacrifice for ___?
8. How much would you be willing to give up to benefit ___?
9. How far would you go out of your way to do something for ___?
10. How easily could you accept not helping ___?

**Full 7-item Relationship Satisfaction scale:**

Citation: Hendrick, S. S. (1988). A generic measure of relationship satisfaction. *Journal of Marriage and the Family*, 93-98.

Instructions: The sentences below reflect different aspects of your relationship. Please use the scales below to indicate how satisfied you are with your relationship in regards to each sentence.

Please use the following scale

| 1 | 2 | 3 | 4 | 5 | 6 | 7 |
| --- | --- | --- | --- | --- | --- | --- |
| Strongly Disagree |  |  | Neither agree nor disagree |  |  | Strongly Agree |

1. How well does your partner meet your needs?
2. In general, how satisfied are you with your relationship?
3. How good is your relationship compared to most?
4. To what extent has your relationship met your original expectations?

Please use the following scale

| \| 1 \| 2 \| 3 \| 4 \| 5 \| 6 \| 7 \| \| --- \| --- \| --- \| --- \| --- \| --- \| --- \| \| Never \|  \|  \| Sometimes \|  \|  \| Very Often \| |
| --- | --- | --- | --- | --- | --- | --- | --- | --- | --- | --- | --- | --- | --- | --- |

1. How often do you wish you had not gotten into this relationship?

Please use the following scale

| \| 1 \| 2 \| 3 \| 4 \| 5 \| 6 \| 7 \| \| --- \| --- \| --- \| --- \| --- \| --- \| --- \| \| Not at All \|  \|  \| Somewhat \|  \|  \| A Great Deal \| |
| --- | --- | --- | --- | --- | --- | --- | --- | --- | --- | --- | --- | --- | --- | --- |

1. How much do you love your partner?

Please use the following scale

| 1 | 2 | 3 | 4 | 5 | 6 | 7 |
| --- | --- | --- | --- | --- | --- | --- |
| None |  |  | Some |  |  | Very Many |

1. How many problems are there in your relationship?

**Study 3: Day-To-Day Variability in Perceptions of Responsiveness and Affectionate Touch**

**Experimental Manipulation**

Critically, the hypotheses in the current study are about daily associations between variables, capitalizing on the quasi-experimental nature of daily data as a strength of the method, and we had no reason to predict that the in-lab manipulation would further influence the strength of those associations, so we do not take condition into account other than to use it as a control variable in analyses and to conduct an exploratory test of whether condition moderates effects (results are reported in OSM).

Upon arrival to the lab, couples were randomly assigned to either an expressed gratitude condition or active control condition. Based on the condition, all couples received instructions about a topic they were going to discuss and then each couple member considered what they would say. Then they each took turns discussing their topic of choice. When in the role of “respondent,” participants were explicitly told, “when your partner is telling you about the details of his/her day, you can respond to, add to, or talk about as much or as little as you would under normal circumstances.”

**Full experimental conditions:**

Those in the expressed gratitude condition received these instructions:

We are interested in how couples talk about the kind things they do for one another. We are interested in hearing about specific things. We’d like you to think about a specific positive thing your partner did for you recently for which you felt grateful. Your partner’s positive gesture may be something that happened before but continues to make you grateful, or something going on now. Some examples would be helping to solve a problem, surprising you with a gift, taking time to listen to a concern, spending time doing something he or she would not typically do, or similar things. We’d like you to pick something good that has been on your mind recently, no matter how big or small. We will ask you to thank your partner for his or her kind gesture in your interaction.

Couples in the active control condition received these instructions:

We are interested in how couples talk about the things that happen to them during the course of their day. We are interested in hearing about events that your partner did not participate in or witness. In particular, please focus on the seemingly ordinary events of your day.

**Daily Measures**

Participants – both members of the couple – independently completed 28 nights of brief online reports following the in-lab visit.

***Perceived Partner Responsiveness***

Participants reported on their partner’s responsiveness that day with one item (“I felt that my partner responded to my needs/wishes”) on a 5-point scale with anchors 1 (*very little or not at* *all*) to 5 (*very much*), *M*=4.08 (*SD* at the day, individual, and couple level are 0.75, 0.43, and 0.34, respectively). Across the submitted 2181 nightly reports, 36 responses for this item were missing, or a 98.35% completion rate.

***Affectionate Touch***

Participants reported on three items about their affectionate touch in the past 24 hours with their partner, selected from the general affectionate touch measure reported in the IDA (Light, Grewen, & Amico, 2005; fewer were used for brevity in the nightly report). Specifically, we asked how often (0 = *not in the last 24 hours*, 1 = *once in the last 24 hours,* 2 = *several times in the last 24 hours*, 3 = *five or more times in the last 24 hours*) they held hands; gave each other neck rubs, back massages or any other warm touching activities; and gave hugs lasting for more than a few seconds. Twenty responses to this item were missing, leaving 99.08% compliance.

***Sexual Intercourse***

Participants reported how often they had engaged in sexual intercourse that day, “including any actions that you, as a couple, consider sex.” The single item was measured on the same scale the affectionate touch items (0 *= not in the last 24 hours* to 3 = *five or more times in the last 24 hours*). Thirty-two responses to this item were missing (a 98.35% compliance rate).

**Full affectionate touch measure, including sexual intercourse item:**

| 0 | 1 | 2 | 3 |
| --- | --- | --- | --- |
| Not in the last 24 hours | Once in the last 24 hours | Several times in the last 24 hours | Five or more times in the last 24 hours |

1. How often did you hold hands with your spouse/partner?
2. How often did you give each other neck rubs, back massages or any other warm

touching activities?

1. How often did you give your spouse/partner hugs lasting for more than a few seconds?

**Sexual intercourse item, assessed using the same scale as affectionate touch items:**

1. How often did you and your partner have sex (include any actions that you, as a couple, consider sex)?

**Testing Alternative Explanations: Daily Relationship Satisfaction**

**Single-item daily relationship satisfaction measure:**

1. Today our relationship was: (please choose a number)

| 1 | 2 | 3 | 4 | 5 | 6 | 7 | 8 | 9 |
| --- | --- | --- | --- | --- | --- | --- | --- | --- |
| Terrible |  |  |  | O.K. |  |  |  | Terrific |

1. In one video, it was ambiguous as to whether the target or expresser initiated the kiss, but this observation was retained to keep as much data as possible. Results do not change if video was recoded as 0. [↑](#footnote-ref-1)
