# Supplementary material for: Perceived Partner Responsiveness Forecasts Behavioral Intimacy as Measured by Affectionate Touch
Source: Pers Soc Psychol Bull. 2021 Mar 19;48(2):203–21. doi: 10.1177/0146167221993349 (PMC8801651; doi:10.1177/0146167221993349)
Supplement: sj-docx-2-psp-10.1177_0146167221993349 – Supplemental material for Perceived Partner Responsiveness Forecasts Behavioral Intimacy as Measured by Affectionate Touch [file sj-docx-2-psp-10.1177_0146167221993349.docx]

**Online Supplementary Materials Table of Contents**

Full Analytic Strategy for Preliminary Study (Integrative Data Analysis) ………………...…...59

Participant Information for Sample D from the Preliminary Study .……………………………60

Procedure for Spontaneously-Reported Affectionate Behavior Code in Study 1…………….…61

Procedure for Sexual Intercourse Code in Study 1……………………………………………...62

Results Controlling for Anxious and Avoidant Attachment in Study 1…………………….…..63

Procedure for “Pilot Testing” Task in Study 2 ..….………………………………………….....64

Using the Affectionate Touch Rating Scale as the Measure of Behavior in Study 2 ..………....65

Results of Analyses Including Outliers for Perceived Partner Responsiveness in Study 2 .…...66

Results without Controlling for Expresser Engagement in Study 2…………………………….69

Results Controlling for Communal Strength in Study 2……………………………………..…70

Results Controlling for Relationship Satisfaction in Study 2…………………..…………….....71

Results Controlling for Sexual Intercourse in Study 3 ..…………………………………..…....72

Results Controlling for Daily Relationship Satisfaction in Study 3…………………………….73

Results of Contemporaneous Hypothesis 2 in Study 3………………………………………....76

Moderation Results in Study 3: Exploratory Test of Effect of Condition on

Hypothesis 1 and 2 ……..……………………………………………………………………....77

**Full Analytic Strategy for Preliminary Study (Integrative Data Analysis)**

We conducted an integrative data analysis to test the association between general perceived partner responsiveness and frequency of affectionate touch in the past month in four unique dyadic samples. Because we were not investigating the differences between data source, we harmonized the variables by standardizing them within their respective studies before integrating. Although this standardization removed the mean differences in the variables between samples, it conserved the strength of the targeted association between perceived partner responsiveness and affectionate touch within each sample and, thus, did not influence the current investigation. We also treated data source not as a level of random sampling but as a fixed effect at the second- (or couple)-level (estimating a three-level model with a sample-level random slope failed to converge). Subsequently, we fit the merged data to a two-level (i.e., individual nested within couple) random-intercept model where affectionate touch was predicted by perceived partner responsiveness as a continuous predictor, data source as a 4-factor (i.e., Sample A to D) predictor, and the interaction between data source and perceived partner responsiveness.^[[1]](#footnote-1)^ By setting up the model this way, we harmonized the pooled datasets not only by the scales of variables but also in the association between constructs of interest. We note that this is one of the most simplified versions of Integrative Data Analysis (Curran & Hussong, 2009), which was facilitated by the standard measurement approach and samples within our lab.

**Participant Information for Sample D from the Preliminary Study**

**(Integrative Data Analysis)**

In Sample D, the 272 participants had been together with the partner for about 4.17 years, (*SD* = 5.16) with the majority of the total sample living together (63.6%) and in an exclusive, committed relationship, i.e., married (26.8 %), engaged (10.3%), or dating exclusively (61.0%). The average participant was 27.07 years old (SD = 9.85, range = 55). Participants self-identified as White/Caucasian (65.4%), Black/African-American (11.0%), East Asian (4.8%), South Asian (2.6%), American Indian or Alaskan Native (0.4%), or Other (9.2%); 8.5% of the sample identified as Hispanic. Data from this larger study have been reported in (Citation Blinded).

**Procedure for Spontaneously-Reported Affectionate Behavior Code in Study 1**

The coder followed these instructions when coding the nightly responses for affectionate touch behavior:

Affectionate touch is warm physical contact that communicates fondness and positive regard, as well as love and support. It can take many forms, but some instantiations are hugging, kissing, massaging, and cuddling, and some less obvious versions may be sitting close together or lying down close together doing leisure activities, sleeping/spending the night together or dancing.^[[2]](#footnote-2)^ Further, based on context, there also may be euphemisms of affectionate touch – those count too.

**Procedure for Sexual Intercourse Code in Study 1**

Participant’s nightly responses describing an interaction with their partner that “made the biggest impression today” were also coded for the absence (coded 0) or presence (coded 1) of sexual intercourse (or “get intimate” or other references to sexual activity).

Six independent judges were trained to code the nightly data. All six coders were trained

on the behavioral codes, and during the several weeks of practice they met with the first author

frequently to discuss and recalibrate the ratings. During practice, coders independently worked

through the same 400 responses to establish reliability (21.5% of the total nightly responses in

the study). Then, the remaining 1460 responses were divided among the six raters, where every

response was coded by three raters (i.e., each of the six raters coded a different third of the

dataset). Throughout, all coders were unaware of the hypothesis, and a sensitivity analysis reveal

the data are sensitive to detect a small effect |r| > .07 at power = 80% assuming no

interdependence among data.

Coder reliability was high (all ICCs were at least .85). In the 1860 responses, there were 44 responses that mentioned sexual activity or intercourse.

**Results Controlling for Secure Attachment in Study 1**

To test the possibility that a dispositional third variable was explaining the hypothesized effects in Study 1, we controlled for secure attachment, which was measured at baseline, like general perceptions of partner responsiveness. Perceived partner responsiveness was negatively corelated with attachment avoidance (*r* = -.24, *p* <.01) and attachment anxiety (*r* = -.18, *p* <.05). Both attachment dimensions were entered into the model simultaneously to account for possible covariance (*r* = .28, *p* <.01). We did not find that anxious attachment was associated with spontaneously-reported affectionate touch behavior, *B* = -0.001, *SE* = 0.01, *df* = 961.13, *t* = -0.09, *p* = .93, CI_95%_ = [-0.02, 0.02], nor did we find avoidant attachment was associated with everyday affectionate touch, *B* = -0.002, *SE* = 0.01, *df* = 567.62, *t* = -0.12, *p* = .91, CI_95%_ = [-0.03, 0.02], but perceived partner responsiveness remained significantly associated with everyday affectionate touch, *B* = 0.04, *SE* = 0.02, *df* = 430.49, *t* = 2.02, *p* = .04, CI_95%_ = [0.001, 0.07] when included in the same model.

**Procedure for “Pilot Testing” Task in Study 2**

The experimenter requested that, given the gap in time before the next couple-based task, the Target go to a desk in a room across the lab suite from the primary room to play and rate a computer-based video game that the experimenter said the lab was testing for use in a future study; the video game was pinball and the reason given for needing the Target’s (as opposed to the Expresser’s) ratings was his/her gender (i.e., the experimenter told the Target, “we need more ratings by men/women”). The items to be rated were clearly shown on a piece of paper the experimenter placed next to the computer so the Target could know in advance this would not be a time-consuming assessment. The four ratings assessed how interesting, exciting and challenging participants found the game, and if they would recommend it to others. They were told that as soon as they had played long enough to be able to give those ratings, they could go back to the room where their partner was.

**Using the Affectionate Touch Rating Scale as the Measure of Behavior in Study 2**

In Study 2, the coders also *rated* the video for overall quantity of affectionate touch demonstrated from the Target to the partner across the entire interaction, using the prompt, “how much affectionate touch did the newcomer^[[3]](#footnote-3)^ demonstrate? I.e., warm physical contact that communicates fondness, positive regard, love and support” on a scale from 1 (*none at all*) to 5 (*a great deal*), *M* = 2.04, *SD* = 0.98, ICC = .84. Ratings were averaged across the two coders to create a composite. Rated affectionate touch was correlated with the primary variables in Study 2 as follows: self-reported perceived partner responsiveness following conversation (*r* = .20, *p* < .05); coded target affectionate touch of partner (count, *r* = .93, *p* < .01); coded kisses (*r* = .72, *p* < .01); coded expresser engagement (*r* = .03, *p* = *ns*); baseline self-reported frequency of affectionate touch (*r* = .30, *p* < .01).

The results for affectionate touch rating were consistent with affectionate touch count results documented in the main text. Perceived partner responsiveness was positively associated with higher global ratings of touch, *β* = 0.39, *SE =* 0.16, *t* = 2.43, *p* = 0.017, CI_95%_ = [0.07, 0.71]; the rating of affectionate touch was also not significantly predicted by condition, *β* = 0.22, *SE =* 0.20, *t* = 1.10, *p* = 0.28, CI_95%_ = [-0.17, 0.6] or Expresser engagement, *β* = -0.21, *SE =* 0.14, *t* = -1.44, *p* = 0.15, CI_95%_ = [-0.5, 0.08], which were included in the same model.

**Results of Analyses Including Outliers for Perceived Partner Responsiveness in Study 2**

As documented in a prior publication (Algoe, Kurtz & Hilaire, 2017), three participants had exceptionally low scores on the independent variable in Study 2, perceived partner responsiveness following the expressed gratitude interaction in-lab. In addition to being statistical outliers by conventional standards (i.e., > 3SD below the mean) and non-normally distributed residuals, the notable 1.2 point gap in the otherwise continuous measure between the highest of these scores, which were 2.00, 2.30, and 2.40, and the low end of the rest of the distribution of data – which ranged from 3.60 to 6.00 -- influences assumptions of measurement underlying the regression; additionally, also as noted in the prior publication, in this situation (i.e., satisfied couples having a very positive social interaction) there is something conceptually different about these participants who gave such low ratings as to not even reach the midpoint of the scale. We excluded these three participants from analyses presented in the main text (consistent with the prior publication), and here present the results with these three participants included.

**Supplementary Figure 1.** Distribution of Post-Interaction Perceived Partner Responsiveness Ratings Including Outliers


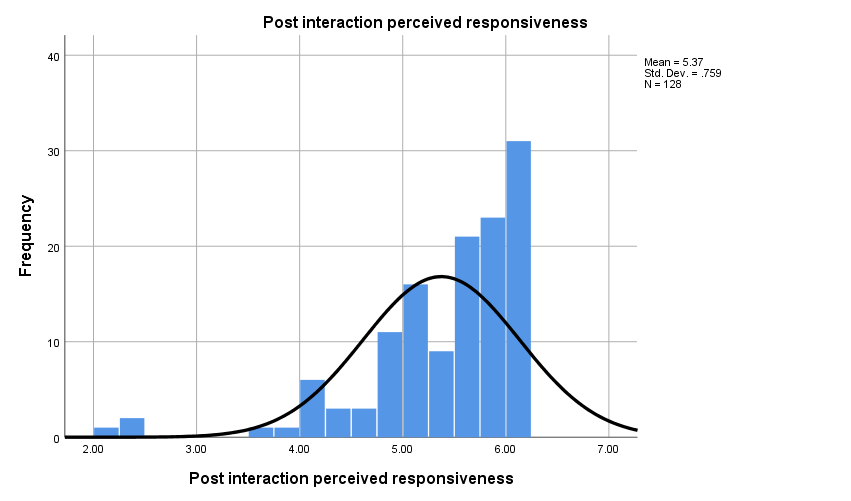


A linear regression using bootstrapped (1,000 repetitions) estimates of the confidence interval was conducted to test the hypothesis for affectionate touch count coding using the entire sample of participants (i.e., including those with outlying perceived partner responsiveness scores). Although trending in the predicted direction, perceived partner responsiveness was no longer a significant predictor of the amount of affectionate touch in the interaction when including the three outliers, *B* = 1.46, *SE =* 0.94, *t* = 1.74, *p* = .10, bootstrapped CI_95%_ = [-0.25, 3.54]) in a model that also controlled for Expresser engagement, *B* = 0.22, *SE* = 1.00, *t =* .23, *p* = .84, bootstrapped CI_95%_ = [-1.84, 2.04] and experimental condition, *B* = -0.01, *SE =* 1.29, *t* = -0.01, *p* = 0.99, bootstrapped CI_95%_ = [-2.56, 2.38]).

For kissing behavior, with outliers included, we also found the association between standardized perceived partner responsiveness in-lab and likelihood of kissing approaching but not reaching statistical significance, *B* = 0.88, *SE* = .46, *df* = 1, *p* = .05, *OR* = 2.41, CI_95%_ = [0.99, 5.89], controlling for Expresser engagement, *B* = 0.59, *SE* = .34, *df* = 1, *p* = .08, *OR* = 1.80, CI_95%_ = [0.92, 3.50] and experimental condition, *B* = 0.18, *SE* = .45, *df* = 1, *p* = .68, *OR* = 1.20, CI_95%_ = [0.50, 2.91].

**Results without Controlling for Expresser Engagement in Study 2**

We removed Expresser engagement from the model and conducted a linear regression with bootstrapped estimates (1000 iterations) of confidence intervals using perceived Expresser responsiveness and condition to predict affectionate touch behavior. Perceived partner responsiveness significantly predicted affectionate touch count code, *b* = .21, *B* = 2.53, *SE =* 1.09, *t* = 2.31, *p* = .01, bootstrapped CI_95%_ = [0.67, 4.50] and experimental condition remained non-significant, *b* = .03, *B* = 0.45, *SE =* 1.29, *t* = 0.35, *p* = 0.74, bootstrapped CI_95%_ = [-2.29, 2.82].

We ran a logistic regression to predict kissing from standardized perceived Expresser responsiveness and experimental condition. Our hypothesis held that perceived responsiveness would be significantly associated with kissing, *B* = 1.07, *SE* = .38, *df* = 1, *p* = .005, *OR* = 2.92, CI_95%_ = [1.39, 6.12], even controlling for condition, which was not a significant predictor, *B* = 0.44, *SE* = .47, *df* = 1, *p* = .34, *OR* = 1.56, CI_95%_ = [0.62, 3.88].

The perceived responsiveness scale was standardized, meaning the kissing finding is interpreted thus: a one-standard deviation increase in the Target’s perception of Expresser’s responsiveness was associated with a 292% increase in the likelihood of kissing the Expresser in the next interaction.

**Results Controlling for Communal Strength in Study 2**

We tested communal strength as an alternative explanation for our findings by controlling for dispositional communal strength measured at baseline alongside perceptions of Expresser responsiveness during the gratitude expression to predict Target’s affectionate touch in the laboratory. In a linear regression using bootstrapped (1,000 repetitions) estimates of the confidence interval, communal strength was not associated with affectionate touch behavior, *b* = 0.04, *B* = 0.31, *SE* = 0.88, *t =* 0.35, *p* = .74, bootstrapped CI_95%_ = [-1.34, 2.07], but perceived responsiveness remained a significant predictor of Target’s affectionate touch, *b* = 0.20, *B* = 2.34, *SE* = 1.27, *t =* 1.85, *p* = .03, bootstrapped CI_95%_ = [0.27, 4.59]; affectionate touch continued to not be predicted by Expresser engagement, *b* = -0.02, *B* = -0.25, *SE* = 0.99, *t = -*0.25, *p* = .81, bootstrapped CI_95%_ = [-1.34, 1.76], or experimental condition, *b* = 0.03, *B* = 0.47, *SE* = 1.35, *t =* 0.35 , *p* = .73, bootstrapped CI_95%_ = [-2.15, 2.99] when included in this same model.

A multiple logistic regression model showed that general communal strength did not predict a greater likelihood of kissing, *β* = -0.51, *SE* = 0.33, *df* = 1, *p* = .12, *OR* = 0.60, CI_95%_ = [0.31, 1.14], but standardized perceived Expresser responsiveness still predicted a greater likelihood of kissing during private leisure time, *β* = 1.38, *SE* = 0.45, *df* = 1, *p* = .002, *OR* = 3.98, CI_95%_ = [1.64, 9.63]; kissing was not significantly predicted by Expresser engagement, *β* = 0.55, *SE* = 0.35, *df* = 1, *p* = .12, *OR* = 1.73, CI_95%_ = [0.87, 3.44], or by condition, *β* = 0.32, *SE* = 0.49, *df* = 1, *p* = .51, *OR* = 1.38, CI_95%_ = [0.53, 3.57], when included in the same model.

**Results Controlling for Relationship Satisfaction in Study 2**

We also tested global relationship satisfaction as an alternative explanation for our findings. Bootstrapping estimates of confidence intervals with 1,000 repetitions, relationship satisfaction did not predict affectionate touch behavior, *b* = -0.64, *B* = 1.50, *SE* = -0.04, *t = -*0.43, *p* = .67, bootstrapped CI_95%_ = [-3.53, 2.31], but perceived responsiveness remained a significant predictor of Target’s affectionate touch on the couch, *b* = 0.23, *B* = 2.77, *SE* = 1.23, *t =* 1.85, *p* = .008, bootstrapped CI_95%_ = [0.70, 4.87]; Expresser engagement, *b* = -0.02, *B* = -0.23, *SE* = 0.99, *t = -*0.23, *p* = .83, bootstrapped CI_95%_ = [-2.34, 1.81], and experimental condition, *b* = 0.03, *B* = 0.38, *SE* = 1.33, *t =* 0.28 , *p* = .78, bootstrapped CI_95%_ = [-2.25, 3.01] were not significant covariates.

A multiple logistic regression model showed that general relationship satisfaction did not predict a greater likelihood of kissing, *β* = 0.23, *SE* = 0.55, *df* = 1, *p* = .68, *OR* = 1.25, CI_95%_ = [0.43, 3.66], but standardized Expresser responsiveness perceived during the gratitude expression still predicted a greater likelihood of kissing during private leisure time, *β* = 1.06, *SE* = 0.42, *df* = 1, *p* = .012, *OR* = 2.88, CI_95%_ = [1.26, 6.56]; kissing was associated with neither Expresser engagement, *β* = 0.54, *SE* = 0.35, *df* = 1, *p* = .13, *OR* = 1.71, CI_95%_ = [0.86, 3.41], nor condition, *β* = 0.42, *SE* = 0.48, *df* = 1, *p* = .38, *OR* = 1.51, CI_95%_ = [0.60, 3.84], when included in the same model.

**Results Controlling for Sexual Intercourse in Study 3**

To investigate the possibility that daily sexual intercourse may be driving the associations found in Study 3, we conducted the same analyses while controlling for the sexual intercourse item to further rule out the possibility that non-sexual affectionate touch (e.g., hug) is a mere downstream consequence of sexual intercourse. We present full results for both Hypotheses in Supplementary Table 1. The hypothesized conclusions remain the same and are independent of this effect.

**Supplementary Table 1.** Test of Hypothesis 1 and 2 Using Disaggregated Within- and Between-Person Variance *Including* Controlling for Daily Sexual Intercourse

|  |  |  |  |  | 95% CI | |  |
| --- | --- | --- | --- | --- | --- | --- | --- |
|  | B | SE | df | t | Low | High |  |
| Hypothesis 1: perceived partner responsiveness🡪 same day affectionate touch | | | | | |  |  |
| Within-person deviations of perceived partner responsiveness | 0.02 | 0.02 | 1675.57 | 8.51*** | 0.10 | 0.17 |  |
| Prior day affectionate touch | 0.37 | 0.02 | 1720.46 | 17.85*** | 0.33 | 0.41 |  |
| Between-individual differences in perceived partner responsiveness | 0.17 | 0.06 | 73.34 | 2.82** | 0.05 | 0.29 |  |
| Experimental condition | -0.04 | 0.08 | 39.81 | -0.53 | -0.21 | 0.12 |  |
| Sexual intercourse | 0.26 | 0.03 | 1719.60 | 10.28*** | 0.21 | 0.32 |  |
| Hypothesis 2: affectionate touch🡪 next day perceptions of touch giver’s responsiveness | | | | | | | |
| Within-person deviations of affectionate touch | 0.08 | 0.04 | 1577.83 | 2.06* | 0.00 | 0.15 |  |
| Present day perceptions of touch giver’s responsiveness | 0.15 | 0.03 | 1628.12 | 5.92*** | 0.10 | 0.20 |  |
| Between-individual differences in affectionate touch | 0.11 | 0.10 | 76.08 | 1.09 | -0.90 | 0.32 |  |
| Experimental condition | 0.05 | 0.13 | 40.45 | 0.40 | -0.20 | 0.29 |  |
| Sexual intercourse | -0.04 | 0.21 | 1602.88 | -0.49 | -0.10 | 0.60 |  |

**p* < .05. ***p* < .01 ****p* < .001. CI = confidence interval.

**Results Controlling for Daily Relationship Satisfaction in Study 3**

For Hypothesis 1, we attempted to rule out a situational alternative explanation, which is that increases in daily relationship satisfaction explain the effects on both perceived partner responsiveness and affectionate touch. Further, Hypothesis 2 could be alternatively explained by relationship satisfaction, such that one’s satisfaction drove both their own affectionate touch and their partner’s perception that they are more responsive the next day, *or* perhaps the partner’s perception of the participant’s responsiveness was driven by their own relationship satisfaction that day.

We tested these situational alternative explanations in Hypothesis 1 (contemporaneous and lagged) and present results in Supplementary Table 2. For both versions of the daily satisfaction control in Hypothesis 2 (participant’s relationship satisfaction or partner’s), see Supplementary Table 3. In all analyses, we disaggregated between- and within-person effects.

**Supplementary Table 2.** Test of Hypothesis 1 and Hypothesis 1 Lagged Using Disaggregated Within- and Between-Person Variance *Controlling* for Daily Relationship Satisfaction

|  |  |  |  |  | 95% CI | |  |
| --- | --- | --- | --- | --- | --- | --- | --- |
|  | B | SE | df | t | Low | High |  |
| Hypothesis 1: perceived partner responsiveness🡪 same day affectionate touch | | | | | |  |  |
| Within-person deviations of perceived partner responsiveness | 0.08 | 0.02 | 1708.70 | 3.95*** | 0.04 | 0.12 |  |
| Prior day affectionate touch | 0.37 | 0.02 | 1723.42 | 17.91*** | 0.33 | 0.42 |  |
| Between-individual differences in perceived partner responsiveness | 0.07 | 0.06 | 80.81 | 1.07 | -0.06 | 0.19 |  |
| Experimental condition | -0.03 | 0.09 | 39.67 | -0.39 | -0.21 | 0.14 |  |
| Present day relationship satisfaction | 0.11 | 0.02 | 1761.21 | 6.80*** | 0.08 | 0.14 |  |
| Hypothesis 1 Lagged: perceived partner responsiveness🡪 next day affectionate touch | | | | | | |  |
| Within-person deviations of perceived partner responsiveness | 0.02 | 0.02 | 1720.29 | 0.92 | -0.02 | 0.06 |  |
| Present day affectionate touch | 0.40 | 0.02 | 1603.66 | 17.96*** | 0.36 | 0.45 |  |
| Between-individual differences in perceived partner responsiveness | 0.17 | 0.06 | 76.77 | 2.80** | 0.05 | 0.29 |  |
| Experimental condition | -0.02 | 0.08 | 38.29 | -0.27 | -0.19 | 0.14 |  |
| Prior day relationship satisfaction | -0.01 | 0.02 | 1770.89 | -0.87 | -0.04 | 0.02 |  |

**p* < .05. ***p* < .01 ****p* < .001. CI = confidence interval; PPR = perceptions of partner’s responsiveness; AT = affectionate touch; Exp condition = experimental condition.

**Supplementary Table 3.** Test of Hypothesis 2 Using Disaggregated Within- and Between-Person Variance *Controlling* for 1) Touch Giver’s Relationship Satisfaction or 2) Touch Receiver’s Satisfaction Next Day

|  |  |  |  |  | 95% CI | |  |
| --- | --- | --- | --- | --- | --- | --- | --- |
|  | B | SE | df | t | Low | High |  |
| Hypothesis 2 Version 1: affectionate touch🡪 next day perceptions of touch giver’s responsiveness | | | | | | | |
| Within-person deviations of affectionate touch | 0.06 | 0.04 | 1574.87 | 1.64 | -0.01 | 0.13 |  |
| Present day perceptions of touch giver’s responsiveness | 0.14 | 0.03 | 1532.17 | 5.13*** | 0.08 | 0.19 |  |
| Between-individual differences in affectionate touch | 0.12 | 0.10 | 74.32 | 1.14 | -0.09 | 0.32 |  |
| Experimental condition | 0.04 | 0.12 | 39.56 | 0.31 | -0.21 | 0.28 |  |
| Present day relationship satisfaction of *touch provider* | 0.02 | 0.02 | 1547.85 | 0.98 | -0.02 | 0.06 |  |
| Hypothesis 2 Version 2: affectionate touch🡪 next day perceptions of touch giver’s responsiveness | | | | | | | |
| Within-person deviations of affectionate touch | 0.15 | 0.03 | 1580.04 | 4.30*** | 0.08 | 0.21 |  |
| Present day perceptions of touch giver’s responsiveness | 0.09 | 0.02 | 1643.90 | 3.86*** | 0.04 | 0.13 |  |
| Between-individual differences in affectionate touch | 0.15 | 0.10 | 80.44 | 1.47 | -0.05 | 0.35 |  |
| Experimental condition | -0.01 | 0.11 | 79.22 | -0.08 | -0.22 | 0.20 |  |
| Next day relationship satisfaction of *partner* | 0.28 | 0.02 | 1640.43 | 15.11*** | 0.24 | 0.31 |  |

**p* < .05. ***p* < .01 ****p* < .001. CI = confidence interval; PPR = perceptions of partner’s responsiveness; AT = affectionate touch; Exp condition = experimental condition.

**Results of Contemporaneous Hypothesis 2 in Study 3**

We ran ancillary non-lagged models of Hypothesis 2, using present day affectionate touch to predict perceptions of touch giver’s responsiveness the *same day*, controlling for perceptions of responsiveness the prior day. In Supplementary Table 4, we present results *not* controlling for touch giver’s relationship satisfaction (top panel) and *while* controlling for touch giver’s relationship satisfaction (bottom panel).

**Supplementary Table 4.** Test of Non-Lagged Hypothesis 2 Using Disaggregated Within- and Between-Person Variance, *Not Controlling* and *Controlling* for Daily Relationship Satisfaction

|  |  |  |  |  | 95% CI | |  |
| --- | --- | --- | --- | --- | --- | --- | --- |
|  | B | SE | df | t | Low | High |  |
| Hypothesis 2 Contemporaneous: affectionate touch🡪 same day perceptions of touch giver’s responsiveness | | | | | | |  |
| Within-person deviations of affectionate touch | 0.27 | 0.03 | 1582.53 | 7.78*** | 0.20 | 0.34 |  |
| Prior day perceptions of touch giver’s responsiveness | 0.12 | 0.02 | 1655.19 | 4.96*** | 0.07 | 0.16 |  |
| Between-individual differences in affectionate touch | 0.13 | 0.11 | 77.09 | 1.25 | -0.08 | 0.34 |  |
| Experimental condition | 0.06 | 0.12 | 39.82 | 0.47 | -0.19 | 0.31 |  |
| Hypothesis 2 Contemporaneous: affectionate touch🡪 same day perceptions of touch giver’s responsiveness | | | | | | |  |
| Within-person deviations of affectionate touch | 0.09 | 0.03 | 1597.37 | 3.02** | 0.03 | 0.14 |  |
| Prior day perceptions of touch giver’s responsiveness | 0.07 | 0.02 | 1617.19 | 3.75*** | 0.03 | 0.11 |  |
| Between-individual differences in affectionate touch | 0.11 | 0.07 | 73.19 | 1.56 | -0.30 | 0.24 |  |
| Experimental condition | -0.04 | 0.08 | 40.55 | -0.58 | -0.20 | 0.11 |  |
| Present day relationship satisfaction | 0.47 | 0.02 | 1599.69 | 30.17*** | 0.44 | 0.50 |  |

**p* < .05. ***p* < .01 ****p* < .001. CI = confidence interval; PPR = perceptions of partner’s responsiveness; AT = affectionate touch; Exp condition = experimental condition.

**Moderation Results in Study 3:** **Exploratory Test of Effect of Condition on**

**Hypothesis 1 and 2**

This sample underwent an experimental manipulation during the 28 days of nightly diaries. Because it is possible the manipulation unintentionally moderated the associations found in Study 3, we wanted to test for possible moderation of condition on the association between perceived responsiveness and affectionate touch. Full results including testing for possible moderation for both hypotheses can be found in Supplementary Table 5. They provide no evidence that experimental condition moderated the hypothesized effects presented in the main text.

**Supplementary Table 5.** Test of Hypothesis 1 and 2 Using Disaggregated Within- and Between-Person Variance *Including* Exploratory Test of Moderation by Experimental Condition

|  |  |  |  |  | 95% CI | |  |
| --- | --- | --- | --- | --- | --- | --- | --- |
|  | B | SE | df | t | Low | High |  |
| Hypothesis 1: perceived partner responsiveness🡪 same day affectionate touch | | | | | |  |  |
| Within-person deviations of perceived partner responsiveness | 0.18 | 0.02 | 1676.99 | 8.02*** | 0.14 | 0.23 |  |
| Prior day affectionate touch | 0.38 | 0.02 | 1708.94 | 17.96*** | 0.34 | 0.42 |  |
| Between-individual differences in perceived partner responsiveness | 0.07 | 0.09 | 66.87 | 0.79 | -0.11 | 0.26 |  |
| Experimental condition | -0.65 | 0.50 | 70.05 | -1.29 | -1.65 | 0.35 |  |
| Within-PPR x Exp cond | -0.05 | 0.03 | 1677.92 | -1.57 | -0.11 | 0.01 |  |
| Between-PPR x Exp cond | 0.15 | 0.12 | 69.37 | 1.27 | -0.09 | 0.39 |  |
| Hypothesis 2: affectionate touch🡪 next day perceptions of touch giver’s responsiveness | | | | | | | |
| Within-person deviations of affectionate touch | 0.01 | 0.05 | 1572.43 | 0.13 | -0.09 | 0.11 |  |
| Present day perceptions of touch giver’s responsiveness | 0.14 | 0.02 | 1638.30 | 5.75*** | 0.09 | 0.19 |  |
| Between-individual differences in affectionate touch | 0.09 | 0.15 | 77.69 | 0.62 | -0.20 | 0.39 |  |
| Experimental condition | -0.02 | 0.31 | 68.44 | -0.06 | -0.63 | 0.59 |  |
| Within-AT x Exp cond | 0.13 | 0.07 | 1571.65 | 1.82 | -0.01 | 0.26 |  |
| Between-AT x Exp cond | 0.04 | 0.21 | 74.89 | 0.21 | -0.37 | 0.46 |  |

**p* < .05. ***p* < .01 ****p* < .001. CI = confidence interval; PPR = perceptions of partner’s responsiveness; AT = affectionate touch; Exp condition = experimental condition.

1. We did not include random slopes because, as in all cross-sectional dyadic designs, there were only two samples—partners—within each couple and this made random-slope models saturated and not estimable. [↑](#footnote-ref-1)
2. We note that some of the less obvious affectionate touch behaviors – e.g., dancing, spending the night together – are more obvious when considering that in the romantic couple context there is a high likelihood that those behaviors also included or co-occurred with affectionate touch. This assumption might not be made in other contexts, such as applying the same code to reports from friends or acquaintances. [↑](#footnote-ref-2)
3. For the coders, the Target was labeled the “newcomer” because s/he was always the person entering the room at the beginning of the video. We intentionally kept coders unaware of the study procedures (e.g., the expressed gratitude task) and the roles to which participants were assigned in the prior interaction (i.e., Target), so as not to bias their ratings. [↑](#footnote-ref-3)
